# Genome-wide analysis of the apple CaCA superfamily reveals that MdCAX proteins are involved in the abiotic stress response as calcium transporters

**DOI:** 10.1186/s12870-021-02866-1

**Published:** 2021-02-08

**Authors:** Ke Mao, Jie Yang, Min Wang, Huayu Liu, Xin Guo, Shuang Zhao, Qinglong Dong, Fengwang Ma

**Affiliations:** State Key Laboratory of Crop Stress Biology for Arid Areas/Shaanxi Key Laboratory of Apple, College of Horticulture, Northwest A &F University, Yangling, 712100 Shaanxi China

**Keywords:** Apple, Calcium, CaCA superfamily, Ca^2+^/H^+^ exchangers, Abiotic stress

## Abstract

**Background:**

Calcium (Ca^2+^) plays an important role in plant growth and development, and the maintenance of calcium homeostasis is necessary for the survival of all plant species. Ca^2+^/H^+^ exchangers (CAXs) are a subgroup of the CaCA (Ca^2+^/cation antiporter) superfamily. In general, CAX proteins mediate cytosolic Ca^2+^ entry into vacuoles to prevent excessive accumulation of Ca^2+^ in the cytosol. The CaCA superfamily has been identified and characterised in many plant species; however, characterisation of the CaCA superfamily and functional study of apple CAX proteins have yet to be conducted in apple (*Malus* × *domestica* Borkh.).

**Results:**

Here, we identified 21 CaCA family proteins in apple for the first time. Phylogenetic and gene structure analysis, as well as prediction of conserved motifs, suggested that these proteins could be classified into four groups: CAX, CCX, NCL, and MHX. Expression analysis showed that the 10 *MdCAX* genes we cloned strongly responded to calcium and abiotic stress treatments. Collinearity analysis and characterisation of calcium transport capacity resulted in the identification of a pair of segmental duplication genes: *MdCAX3L-1* and *MdCAX3L-2*; MdCAX3L-2 showed strong calcium transport capacity, whereas MdCAX3L-1 showed no calcium transport capacity. Yeast two-hybrid (Y2H) assays showed that these two proteins could interact with each other. The high sequence similarity (94.6%) makes them a good model for studying the crucial residues and structural basis of the calcium transport of CAX proteins. Prediction of the protein interaction network revealed several proteins that may interact with CAX proteins and play important roles in plant stress responses, such as SOS2, CXIP1, MHX, NRAMP3, and MTP8.

**Conclusions:**

Our analysis indicated that MdCAX proteins have strong calcium transport capacity and are involved in the abiotic stress response in apple. These findings provide new insight and rich resources for future studies of MdCAX proteins in apple.

**Supplementary Information:**

The online version contains supplementary material available at 10.1186/s12870-021-02866-1.

## Background

Calcium (Ca^2+^) is an essential element for plant growth and development. On the one hand, Ca^2+^ is crucial for structural and metabolic needs to maintain plant membrane stability, cell wall stabilization, and cell integrity [1, 2]. On the other hand, Ca^2+^ functions as a second messenger to regulate plant physiological responses and gene expression changes in response to various environmental stimuli [3–7]. All of these functions of calcium depend on the regulation of cytosolic Ca^2+^ concentration, which relies on the coordination of Ca^2+^-permeable ion channels, Ca^2+^-ATPases, and Ca^2+^/H^+^ exchangers (CAXs) [8, 9].

In general, CAX proteins are located in vacuolar membranes and mediate cytosolic Ca^2+^ entry into vacuoles to prevent excessive accumulation of Ca^2+^ in the cytosol [10, 11]. Maintaining a basal Ca^2+^ concentration also helps to prime the generation of cytosolic Ca^2+^ signals [12]. CAX proteins belong to the Ca^2+^/cation antiporter (CaCA) superfamily, which is present in all organisms from bacteria to higher plants and animals [9]. Proteins in this family are about 300 to 1000 amino acid (AA) residues in length and have similar topological structures [9, 13]. These proteins usually facilitate the efflux of Ca^2+^ against a concentration gradient across the membrane and promote the influx of monovalent cations, such as H^+^, Na^+^, or K^+^ in exchange [13, 14]. The CaCA superfamily is divided into five families: the YRBG family, the Na^+^/Ca^2+^ exchanger (NCX) family, the Na^+^/Ca^2+^, K^+^ exchanger (NCKX) family, the cation/Ca^2+^ exchanger (CCX) family, and the CAX family [9, 14]. The YBRG family is present exclusively in prokaryotes, and the NCX and NCKX families are present in animals and algae but are absent in higher plants [13–16]. Only the CCX and CAX families are found in higher plants. Additional studies have described two additional groups of CaCA proteins in land plants named NCX-like (NCL) [17, 18] and Mg^2+^/H^+^ exchanger (MHX) proteins [9, 11, 19].

Since the *CAX* gene was first identified in *Arabidopsis* [20], several studies have shown that CAX proteins are widespread across the tree of life, with the exception of mammals, insects, and nematodes [9, 11, 21]. CAX proteins are divided into three categories: Type I, II, and III [22]. In plants, CAX proteins are classified as Type I CAXs, and they can be divided further into two distinct subgroups: Type I-A and Type I-B [21, 23]. The CAX proteins in the Type I-A subgroup were originally thought to be exclusively involved in Ca^2+^ transport [20, 24]; in contrast, type I-B members can facilitate the transport of several ions, such as Cd^2+^, Zn^2+^, and Mn^2+^ in addition to Ca^2+^ [9, 25, 26]. Subsequent research has shown that type I-A CAX proteins can also facilitate the transport of multiple ions [27–30].

CAX proteins are characterised by 11 transmembrane (TM) helices, named TM1 to TM11 [23]. The protein sequences of these TM regions are highly conserved in all plant species, with most variations occurring in the loop and N- and C-terminal regions [21]. Among these TMs, TM2 to TM11 helices are separated by a cytosolic loop (acidic helix) into two weakly homologous parts (TM2–TM6 and TM7–TM11), and this forms the core structure for Ca^2+^ transport [11]. Within TM 2–3 and 7–8, two highly conserved α-repeat regions are crucial for ion selectivity, binding, and transport [9, 14]. Following the release of the crystal structure of Ca^2+^/H^+^ antiporter protein (ScVCX1) in eukaryotes [31], various key structures and residues related to calcium ion transport have been explored and verified [31]. Combined with ScVCX1, structural studies of CAX_Af and YfkE have indicated that CAX family proteins form homomeric dimers (ScVCX1 and CAX_Af) or trimers (YfkE) [31–33]. Studies in plants have also shown that CAX proteins can form homomeric or heteromeric oligomers, which may be important for the regulation of their functional activity [10, 34, 35].

In addition to regulating calcium homeostasis, CAX proteins also play an important role in regulating plant abiotic stress resistance. In *Arabidopsis* and rice, the transcript level of many *CAX* genes increases or decreases in response to various abiotic stresses [11]. In *Arabidopsis*, *cax1* mutants (*cax1–3* and *cax1–4*) exhibit increased freezing tolerance after cold acclimation, whereas in cotton, the gene responsible for freeze tolerance is *GhCAX3* [11]. Overexpression of *SsCAX1* in *Arabidopsis* leads to increased salt sensitivity of transgenic plants, and this phenotype was also observed when *AtCAX1* was ectopically expressed in tobacco [36, 37]. In contrast, overexpression of *GmCAX1* enhances salt tolerance of transgenic *Arabidopsis* [38].

Apple (*Malus domestica*) is one of the world’s most economically important fruits. Its cultivation and extension are restricted by various abiotic stresses, such as drought, salt and low temperature, which are all related to calcium homeostasis and calcium signalling [3, 10, 11, 21, 23]. Besides, studies in various fruit tree crops have shown that calcium plays an important role in regulating fruit development, ripening, quality, storage and preventing damage to fruit caused by adverse environment [39–44]. The study of calcium transport-related proteins is thus important for the resistance breeding of apple. Here, we identified and characterised 21 proteins from the CaCA superfamily in apple. Specifically, an expression analysis under Ca^2+^ treatment, an ectopic expression assay, and a calcium ion fluorescence staining assay in the yeast mutant k667 were used to study the Ca^2+^ response and Ca^2+^ transport capacity of *MdCAX* genes. The results of the expression analysis results indicated that these *MdCAX* genes respond to various abiotic stress treatments. Based on the protein interaction network prediction and the Y2H assay, we show that MdCAX proteins may form homologous or heterologous dimers by protein interactions. The network also indicated that MdCAX proteins may regulate plant stress resistance by interacting with stress-related proteins, such as SOS2. These results provide rich resources for future studies of MdCAX proteins in apple.

## Results

### Genome-wide identification of proteins belonging to the CaCA superfamily in apple

To identify apple CaCA superfamily genes, the HMM file of the Na_Ca_ex domain (PF01699) was downloaded from the Pfam database and used as a query to screen the apple proteome (GDDH13) with HMMER software (version 3.1b2). Using default filter parameters (E-value < 0.05), we obtained a total of 32 proteins (Additional file 1: Table S1 and Additional file 2). Two of them (MD09G1157400, MD14G1008300) were eliminated because of their short lengths (Additional file 1: Table S1 and Additional file 2) [9]. The remaining 30 proteins, along with the 13 CaCA superfamily proteins in *Arabidopsis*, were used for phylogenetic analysis (Additional file 3: Fig. S1). Most of the 30 proteins clustered with the CaCA superfamily proteins in *Arabidopsis* and comprised four groups: CAX, NCL, CCX, and MHX1. However, there was an extra clade that contained six proteins that did not belong to any group in the CaCA superfamily (Additional file 3: Fig. S1). Because of this finding, as well as a comparison of conserved domains (Na_Ca_ex domain; EF-hand domain) between *Arabidopsis* and apple (Additional file 1: Table S1), these proteins were removed in subsequent analyses. Finally, 21 proteins were identified and confirmed as CaCA superfamily members in apple, which contained 11 CAXs, five CCXs, four NCLs, and one MHX1 (Table 1).

**Table 1:** Characterization of the CaCA family proteins in apple

Group	Gene ID	Name	Length (aa)	Mass Weight (kDa)	Charge at PH 7.0	pI	Genomic Location	Best hits	Name (***Arabidopsis***)
**CAX**	**MD17G1143500**	**MdCAX2L-1**	400 (**433**)	47.77	−10.34	5.42	Chr 17: 12,932,234-12,937,843	AT3G13320.1	CAX2
	**MD09G1157100**	**MdCAX2L-2**	513 (**433**)	47.71	−15.17	5.00	Chr 09: 12,520,620-12,526,155	AT3G13320.1	CAX2
	**MD04G1151600**	**MdCAX3L-1**	450	48.32	−4.81	6.08	Chr 04: 24,048,771-24,052,948	AT3G51860.1	CAX3
	**MD12G1165800**	**MdCAX3L-2**	458 (**450**)	49.53	−0.80	6.81	Chr 12: 24,665,244-24,669,792	AT3G51860.1	CAX3
	**MD03G1070900**	**MdCAX3L-3**	451	49.38	−5.85	5.81	Chr 03: 5,786,105-5,790,084	AT3G51860.1	CAX3
	**MD11G1074900**	**MdCAX3L-4**	453	49.76	−10.04	5.11	Chr 11: 6,348,409-6,352,616	AT3G51860.1	CAX3
	**MD04G1151900**	**MdCAX3L-5**	438	47.73	−9.79	5.31	Chr 04: 24,108,144-24,112,570	AT3G51860.1	CAX3
	**MD11G1041700**	**MdCAX5L-1**	450	49.32	−12.86	4.99	Chr 11: 3,560,349-3,565,335	AT1G55730.2	CAX5
	**MD03G1040300**	**MdCAX5L-2**	447	48.92	−10.03	5.15	Chr 03: 3,192,551–3,197,506	AT1G55730.2	CAX5
	**MD09G1134300**	**MdCAX5L-3**	463	50.50	−11.49	5.33	Chr 09: 10,321,364-10,325,618	AT1G55730.2	CAX5
	**MD17G1123100**	**MdCAX5L-4**	434	47.33	−10.30	5.54	Chr 17: 10,683,015-10,689,101	AT1G55730.2	CAX5
**CCX**	**MD12G1011500**	**MdCCX1**	581	63.37	+ 3.24	7.76	Chr 12: 1,189,557-1,191,302	AT5G17860.1	CCX1
	**MD12G1011800**	**MdCCX2**	571	62.43	+ 10.97	8.43	Chr 12: 1,218,407-1,220,122	AT5G17850.1	CCX2
	**MD15G1219100**	**MdCCX3**	653	71.44	+ 2.51	7.66	Chr 15: 17,708,721-17,711,343	AT1G54115.1	CCX4
	**MD02G1094200**	**MdCCX4**	653	71.57	+ 0.65	7.18	Chr 02: 7,479,499-7,481,507	AT1G54115.1	CCX4
	**MD04G1207600**	**MdCCX5**	546	59.29	+ 3.31	7.86	Chr 04: 29,274,566-29,277,830	AT1G08960.1	CCX5
**MHX1**	**MD03G1288600**	**MdMHX1**	530	59.19	−7.76	5.65	Chr 03: 36,723,856-36,727,511	AT2G47600.1	MHX1
**NCL**	**MD03G1238600**	**MdNCL-1**	586	63.75	−12.20	5.44	Chr 03: 32,378,315-32,388,912	AT1G53210.1	NCL
	**MD03G1238700**	**MdNCL-2**	609	67.07	−15.18	5.22	Chr 03: 32,389,435-32,393,941	AT1G53210.1	NCL
	**MD11G1258400**	**MdNCL-3**	584	63.27	−16.53	4.91	Chr 11: 37,186,082-37,191,361	AT1G53210.1	NCL
	**MD17G1233800**	**MdNCL-4**	585	64.56	−7.42	5.28	Chr 17: 28,245,403-28,250,477	AT1G53210.1	NCL

The sequence lengths of the revised proteins (MdCAX2L-1, MdCAX2L-2 and MdCAX3L-2) were listed in parentheses

Because the EF-hand domain was identified in four MdNCL proteins with Pfam but not in AtNCL (Additional file 1: Table S1), we further analyzed these proteins with the SMART database. AtNCL and the four MdNCL proteins contained two Na_Ca_ex domains and two EF-hand domains (Fig. S2), which further supported the phylogenetic tree (Additional file 3: Fig. S1). Except for MD03G1238700, the other three MdNCL proteins, as well as AtNCL, also had a signal peptide in the N terminus (Additional file 4: Fig. S2). MD03G1238700 contained an additional transmembrane region in the N terminus (Additional file 4: Fig. S2).

### Chromosomal location, gene duplication and characterisation of apple CaCA superfamily proteins

**Fig. 1:**
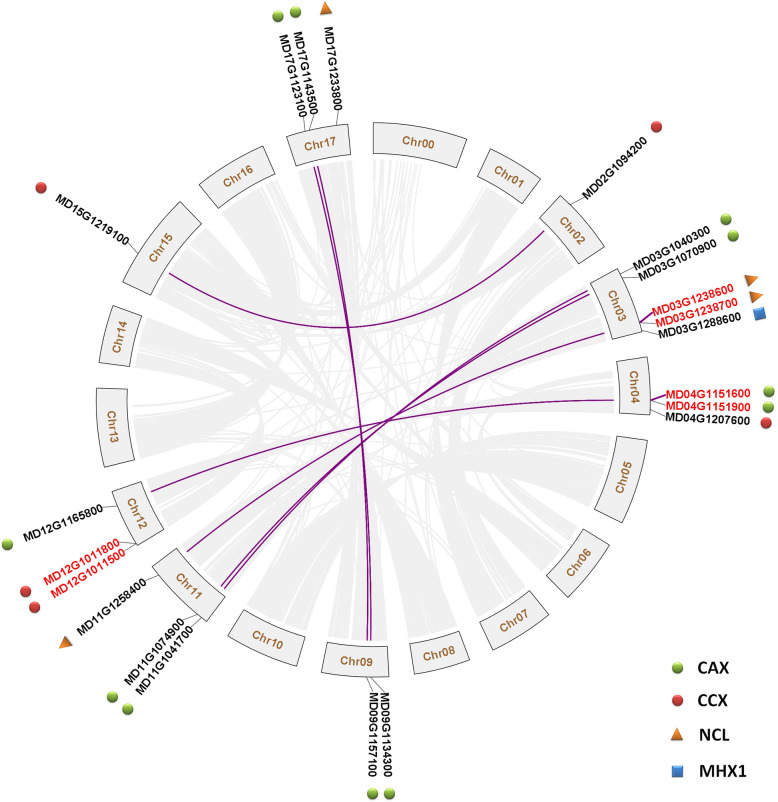
Genome locations of the 21 CaCA family genes in apple. Purple lines and red font indicate the segmental and tandem duplication genes, respectively. Collinear blocks are represented by greyish lines. Chr01 to Chr17 indicate the 17 apple chromosomes, and Chr00 represents the unassembled genomic scaffolds

Segmental and tandem duplications are two of the main causes of gene family expansion in plants [45, 46]. Because the number of CaCA family genes in apple, especially the number of *CAX* and *NCL* genes, was significantly higher than that in *Arabidopsis*, we analyzed the collinear relationship between these CaCA family genes in apple. Based on chromosomal location data (gene_models_20170612.gff3) that were downloaded from the GDR database, these 21 CaCA family genes were mapped to eight apple chromosomes, which ranged from 1 to 5 per chromosome (Fig. 1). Collinear analysis revealed complex patterns of collinearity between different chromosomes, such as collinearity between Chr 2 and Chr 15, Chr 3 and Chr 11, Chr 4 and Chr 12, and Chr 9 and Chr 17 (Fig. 1). This result is consistent with previous research on the apple genome [47, 48]. The collinear analysis also found that 14 CaCA family genes (seven pairs) have undergone segmental duplication events: five pairs of *CAX*, one pair of *CCX* (*MD02G1094200* and *MD15G1219100*), and one pair of *NCL* (*MD03G1238600* and *MD11G1258400*) (Fig. 1). In addition, three pairs of genes underwent tandem duplication events, and CAX (*MD04G1151600* and *MD04G1151900*), CCX (*MD12G1011500* and *MD12G1011800*), and NCL (*MD03G1238600* and *MD03G1238700*) families each contained one pair (Fig. 1).

Using the local blastp method, we identified the orthologs of these 21 apple CaCA family proteins in *Arabidopsis*. These 21 proteins were then named based on the phylogenetic tree (Additional file 3: Fig. S1) and their orthologs in *Arabidopsis* (Table 1). Sequence analysis showed that these proteins were 400 to 653 aa in length, and the CAX family proteins were generally around 450 aa (Table 1). Protein length, mass weight, charge at pH 7.0, isoelectric point (pI), and genomic positions of these CaCA family proteins were summarised in Table 1. Within the CaCA family, CCX proteins were the only group that showed positive values in charge at pH 7.0, and their pI values (> 7.0) were also significantly higher relative to that of other proteins (Table 1).

### Phylogenetic analysis, gene structure display and prediction of the conserved motifs of apple CaCA superfamily proteins

**Fig. 2:**
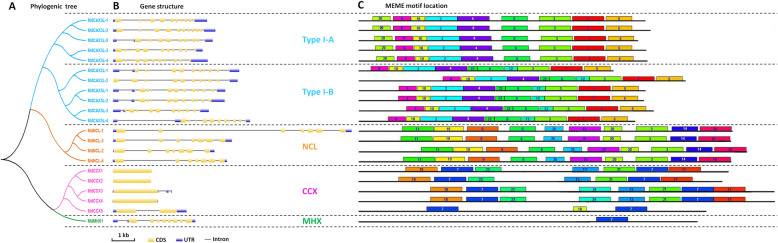
Analysis of phylogenic relationships, gene structure and conserved motifs for CaCA family proteins in apple

To further study the relationship between these 21 CaCA superfamily proteins, we performed detailed phylogenetic, gene structure, and conservative motif analysis (Fig. 2). Phylogenetic analysis showed that these proteins could be classified perfectly into four groups: CAX, NCL, CCX, and MHX [13]. In addition, the CAX family could be divided further into two subgroups—Type I-A and Type I-B (Fig. 2a)—similar to the CAX proteins in other plants [9, 13].

Gene structure analysis also supported this grouping. The gene structure varied greatly between diffferent families (Fig. 2b). Although the length of introns varied, genes that belonged to the same family had the same extron/intron numbers and the same exon-intron composition patterns (Fig. 2b). However, there were two exceptions: *MdCAX3L-5* and *MdCCX5*. Among genes in Type I-A, only *MdCAX3L-5* contained an intron in the 5′ noncoding region, and introns more commonly occurred in 5′ noncoding region exists in Type I-B genes. *MdCCX5* had an intron in the coding region, which made it different from other CCX family genes (Fig. 2b). The gene *MdNCL-1* was also notable for its longer introns, which made its sequence nearly twice as long as other genes in the CaCA superfamily.

In addition to gene structure analysis, the prediction of conserved motifs further supported the phylogenetic grouping. With the online software MEME, a total of 25 conserved motifs was found in these proteins (Fig. 2c and Additional file 5: Fig. S3). Consistent with the phylogenetic tree (Fig. 2a), proteins in the same groups showed similar motif composition patterns, whereas proteins that belonged to different families consisted of different motifs and exhibited completely different composition patterns (Fig. 2c and Additional file 5: Fig. S3). By comparing the motif composition between subgroups Type I-A and Type I-B, we inferred that the three motifs (motif 20, motif 22, and motif 12) could be used to differentiate the two types of CAX proteins (Fig. 2c).

### Gene cloning, sequence alignment, and prediction of the 3D structure of MdCAX proteins

To study the structure and function of CAX family proteins in apple, we cloned the full-length sequences of 10 genes in this family; however, the sequence of one gene (*MdCAX3L-5*) could not be obtained. The results of gene cloning showed that the predicted coding sequences (CDS) of three *MdCAX* genes (*MdCAX2L-1*, *MdCAX2L-2*, and *MdCAX3L-2*) in the apple genome were incorrect (Table 1). The predicted transcription initiation sites (ATG) of *MdCAX2L-1* (*MD17G1143500*) and *MdCAX2L-2* (*MD09G1157100*) were incorrectly shifted backward and forward, respectively, which resulted in the deletion (Additional file 6: Fig. S4a) or addition of an extra sequence (Additional file 6: Fig. S4b) in the N terminus of these two proteins. For *MdCAX3L-2* (*MD12G1165800*), there was an incorrect insertion of four bases in the C terminal region of its coding sequence (Additional file 6: Fig. S4c), which led to the translocation of the coding frame and changes in the amino acid sequence (Additional file 6: Fig. S4d). The CDS and protein sequences of these cloned *MdCAX* genes are listed in Additional file 7, and all of the subsequent analyses were performed using the corrected sequences.

**Fig. 3:**
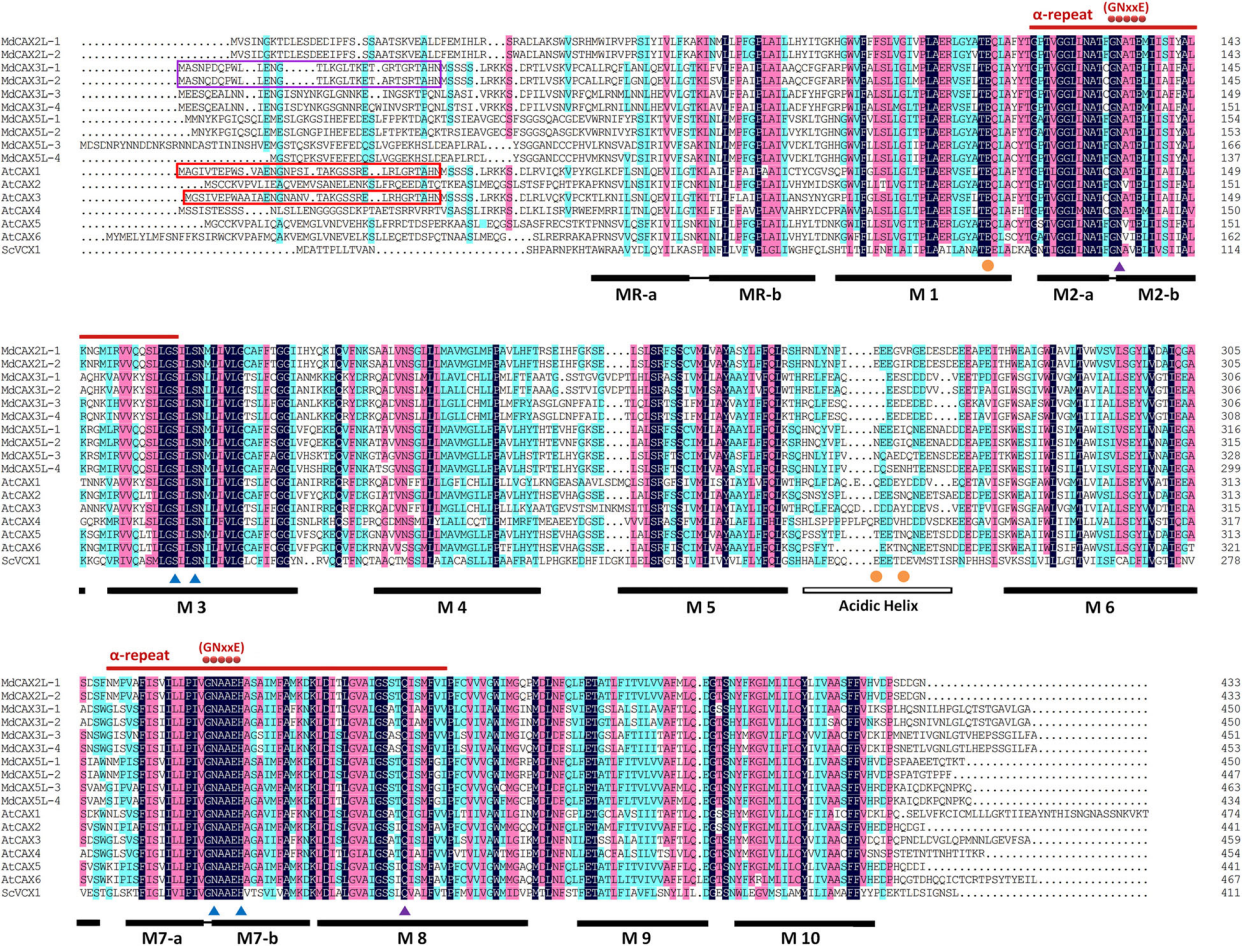
Comparison of the apple MdCAX proteins with AtCAX proteins in *Arabidopsis* and ScVCX1 in yeast. Solid black lines and black box below the sequences indicate the 11 transmembrane regions (MR and M1 to M10) and the acidic helix region, respectively. Red lines and dots above the sequences indicate the two α-repeats and the two “GNxxE” motifs, respectively. Red and purple boxes indicate the N-terminal autoinhibitory region. Yellow dots below the sequences indicate the amino acid residues (Glu 83 of M1, Glu 230 and Asp 234 of the acidic helix) crucial for the coordination of calcium ions in ScVCX1. Purple and blue triangles indicate the residues crucial for the structural maintenance of ScVCX1 between M2 and M8, and between M3 and M7, respectively

Sequence alignment showed that similar to AtCAXs and ScVCX1 proteins, all of the MdCAX proteins contained 11 TM regions (Fig. 3). Except for the first TM helix (MR), the 10 remaining TM regions (M1–M10) were also divided into two halves: TM 2 to 6 and TM 7 to 11, which were linked by an unconserved acidic helix region (Fig. 3). Moreover, there were also two highly conserved cation-binding regions called α-repeat regions, which were located within TM 2–3 and TM 7–8, and each α-repeat contained a “GNxxE” motif (Fig. 3). Based on sequence alignment, we identified the putative N-terminal autoinhibitory domain in MdCAX3L-1 and MdCAX3L-2, which has been found in AtCAX1 and AtCAX3, and was shown to affect calcium ion transport [24, 49] (Fig. 3).

With the release of the 3D structure of the yeast ScVCX1 protein, several crucial residues that were responsible for structural maintenance and ion transport were identified [31]. Based on sequence alignment, we found that these residues also existed and were well conserved in MdCAX proteins (Fig. 3): residues E106 (M2) and E302 (M7) were crucial for Ca^2+^ transport; residues E83 (M1), E230 (acidic helix), and D234 (acidic helix) were responsible for Ca^2+^ concentration; residues N103 (M2) and Q328 (M8) were important for the structural maintenance between M2 and M8; and residues S129 (M3), S132 (M3), N299 (M7), and H303 (M7) were important for the structural maintenance between M3 and M7 [31].

**Fig. 4:**
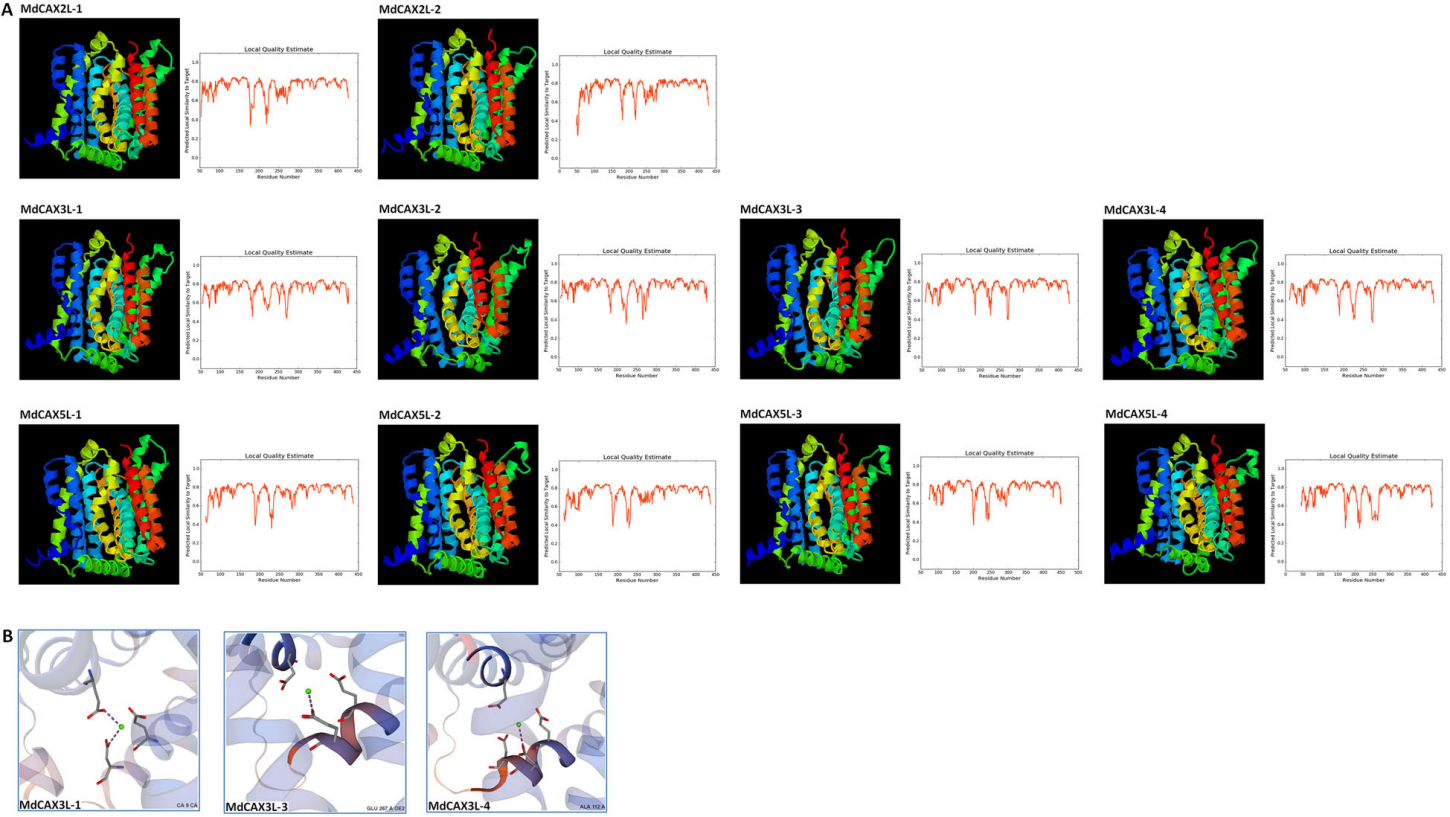
The predicted 3D structure diagram of the CAX family proteins in apple. **a** The diagram of the predicted 3D structure of MdCAX proteins and prediction quality estimate. **b** The residues of MdCAX proteins predicted to be crucial for calcium transport

To more deeply characterise the structure and ion transport function of MdCAX proteins, we predicted their 3D structure based on the structural model of ScVCX1 (PDB ID: 4k1c). As we expected, all of the MdCAX proteins formed perfect spatial structures that were similar to ScVCX1 with local similarity of most residues (coverage: 0.83–0.93) greater than 0.7 (Fig. 4a and Additional file 8: Table S2). Moreover, among these TM regions, M2 and M7 were kinked at their midpoints to form an hourglass-like structure through which ions (H^+^, Ca^2+^) could pass, which led to the separation of M2a/M2b and M7a/M7b (Fig. 4a). The prediction also identified several residues that may be crucial for Ca^2+^ binding in MdCAX3L-1, MdCAX3L-3, and MdCAX3L-4 (Fig. 4b and Additional file 8: Table S2).

### Expression analysis of *MdCAXs* under calcium ion treatment

**Fig. 5:**
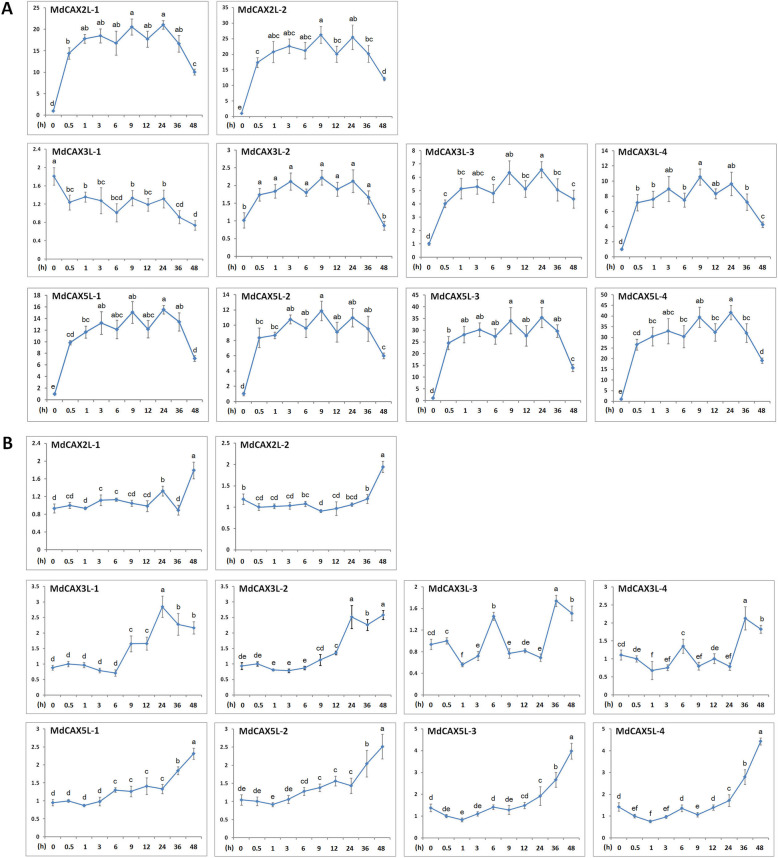
Relative expression analysis of *MdCAX* genes in apple leaves (**a**) or roots (**b**) under calcium ion treatment. The expression level was calculated with respect to control samples (0 h) with the 2^—△△CT^ method. Bars labelled with different letters indicate signigicant differences at *P* < 0.05 based on one-way ANOVA and Duncan’s tests (SPSS software, version 26)

Hydroponic apple seedlings were used to study the response of *MdCAX* genes to calcium ion treatment. *MdCAX* genes responded significantly to calcium ion treatment, especially in apple leaves, with changes in the expression of most *MdCAX* genes more than tenfold (Fig. 5a). In addition, the expression of *MdCAX* genes in leaves was clearly different from that in roots. Under calcium ion treatments, the expression of most *MdCAX* genes showed pattern of initial up-regulation followed by down-regulation in leaves with only one exception, *MdCAX3L-1*, which showed a significant down-regulation expression pattern (Fig. 5a). In contrast, most *MdCAX* genes were continuously up-regulated by Ca^2+^ treatment in roots significantly (Fig. 5b). Among these *MdCAX* genes, *MdCAX3L-1* was also the only one that showed the exact opposite expression pattern in leaves relative to roots (Fig. 5).

### Characterisation of calcium transport activity of CAX proteins in the type I-A subgroup

**Fig. 6:**
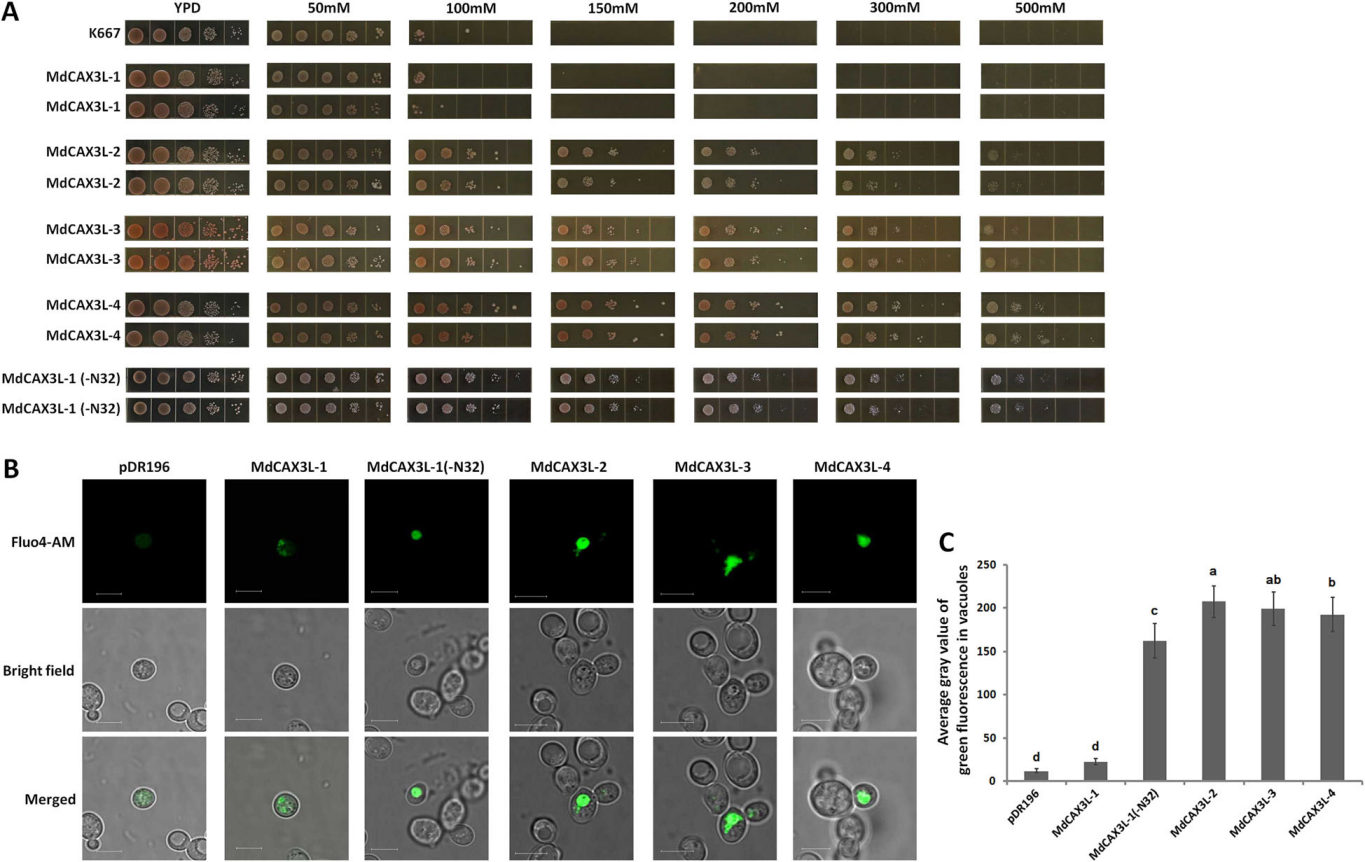
Characterisation of calcium transport activity in apple CAX family proteins. **a** Phenotype of yeast cells treated with different concentrations of calcium ions. The label above the picture shows the concentration of CaCl_2_ uses in the calcium ion treatment. **b** Bright-field and fluorescence microscopy of yeast cells labelled with the calcium-sensitive fluorescent probe Fluo4-AM. The scale bar represents 5 μm. MdCAX3L-1 to MdCAX3L-4 represents the transformation of K667 yeast strains with corresponding full-length *MdCAX* genes, and MdCAX3L-1(−N32) represents the transformation with the truncated *MdCAX3L-1* gene, with 32 amino acids of its N terminus removed. K667 or K667 transformed with the empty vector pDR196 were used as controls. **c** Average grey values of green fluorescence in vacuoles of K667 yeast cells. The gray values of green fluorescence in the vacuoles of ten yeast cells expressing a specified *MdCAX* gene were used to calculate the average grey value of green fluorescence. Bars labelled with different letters indicate signigicant differences at *P* < 0.05 based on one-way ANOVA and Duncan’s tests (SPSS software, version 26)

Based on the calcium ion transport function of CAX1/CAX3 proteins in various plants [2, 20, 29] and the obvious response of *MdCAX* genes to Ca^2+^ treatment, the calcium transport capacity of four *MdCAX3L* genes in Type I-A (Fig. 2) were studied. Firstly, we characterised their calcium transport capacity using the Ca^2+^ sensitive yeast strain K667. In YPD medium, the k667 grew normally (Fig. 6a). However, when the Ca^2+^ concentration increased to 100 mM, its growth was inhibited significantly; when the Ca^2+^ concentration increased further, the strain did not survive at all (Fig. 6a). In contrast, overexpression of any one of the three *MdCAX* genes (*MdCAX3L-2*, *MdCAX3L-3*, and *MdCAX3L-4*) enabled the transgenic strain to survive in the medium with a high concentration of calcium, even when the concentration was increased to > 500 mM (Fig. 6a). This result suggested that these three genes may have strong calcium transport capacity.

Unexpectedly, the overexpression of *MdCAX3L-1*, a segmental duplication gene of *MdCAX3L-2* (Fig. 1) with sequence similarity up to 94.6% (Fig. 3), did not restore the calcium ion-sensitive phenotype of k667 (Fig. 6a). Given the strong inhibitory effect of the N-terminal autoinhibitory region on the calcium transport capacity of CAX proteins [2, 50], we removed this region from MdCAX3L-1 (32aa; Fig. 3) and also transferred the truncated MdCAX3L-1 (−N32) in K667. As expected, the truncated MdCAX3L-1 (−N32) restored the calcium ion-sensitive phenotype of k667 (Fig. 6a), suggesting that the N-terminal region of MdCAX3L-1 may inhibit its calcium transport capacity in a manner similar to AtCAX1 [50].

To further characterise the calcium transport capacity of the four *MdCAX* genes, we carried out the Ca^2+^ fluorescence staining experiment of k667 cells transformed with different *MdCAX* genes using the calcium-sensitive probe Fluo4-AM. Fluorescence observation showed that the k667 yeast cells expressing the truncated *MdCAX3L-1* (−N32) or the three *MdCAX* genes (*MdCAX3L-2*, *MdCAX3L-3*, *MdCAX3L-4*) exhibited strong Fluo4-AM fluorescence in the vacuole, indicating that calcium ions accumulated in large quantities in vacuoles (Fig. 6b, c). In contrast, cells expressing pDR196 or full-length *MdCAX3L-1* exhibited weak fluorescence, and calcium ions did not accumulate in vacuoles (Fig. 6b, c). These results showed that *MdCAX* genes can promote the accumulation of calcium ions in vacuoles, which further demonstrating their function in calcium transport.

### Promoter analysis of *MdCAX* genes

**Fig. 7:**
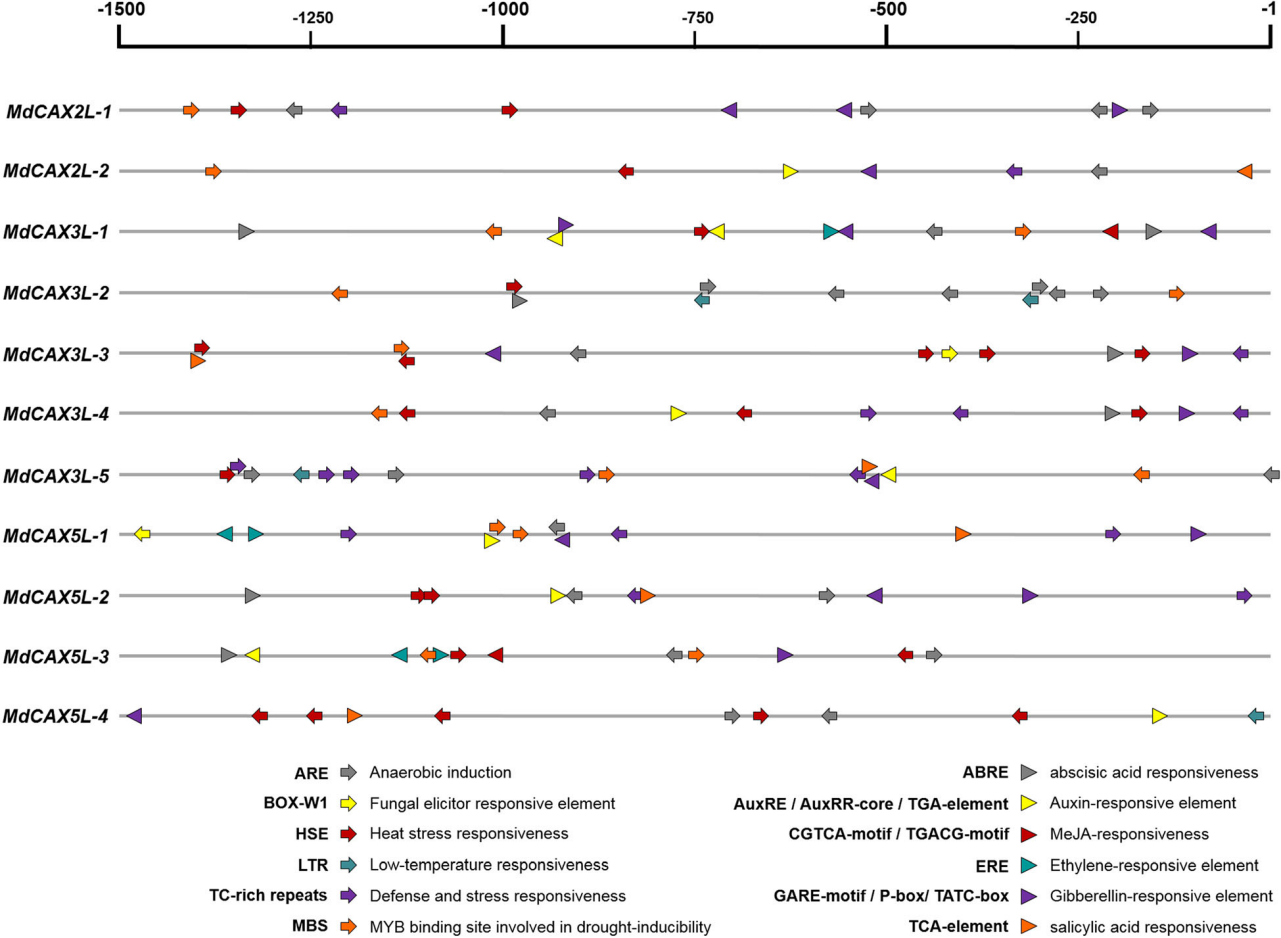
Cis-element analysis of the *MdCAX* genes promoter regions in apple. Positive and negative directions indicate whether the motif exists in the plus or minus strand of the cis-acting elements

Studies in plants have indicated that *CAX* genes respond to various abiotic stresses, such as low temperature, waterlogging, salt, drought and heavy metals [11]. To study the role of *MdCAX* genes in stress responses, we first identified the cis-elements related to the stress response in the promoter regions of these genes. Upstream sequences 1500 bp in length of these *MdCAX* genes (Additional file 9) were obtained and analyzed with the online software PlantCARE. Various cis-elements related to abiotic stress responsiveness, such as hypoxia, heat, low temperature, and drought, and elements related to plant hormones, such as ABA, auxin, MeJA, ethylene, GA, and SA, were found, and many of these cis-elements appeared multiple times (Fig. 7; Additional file 10: Table S3 and Additional file 11: Fig. S5). This result suggested that *MdCAX* genes play an important role in the abiotic stress response and its regulation in apple.

### Expression profile of *MdCAX* genes under abiotic stress treatments

**Fig. 8:**
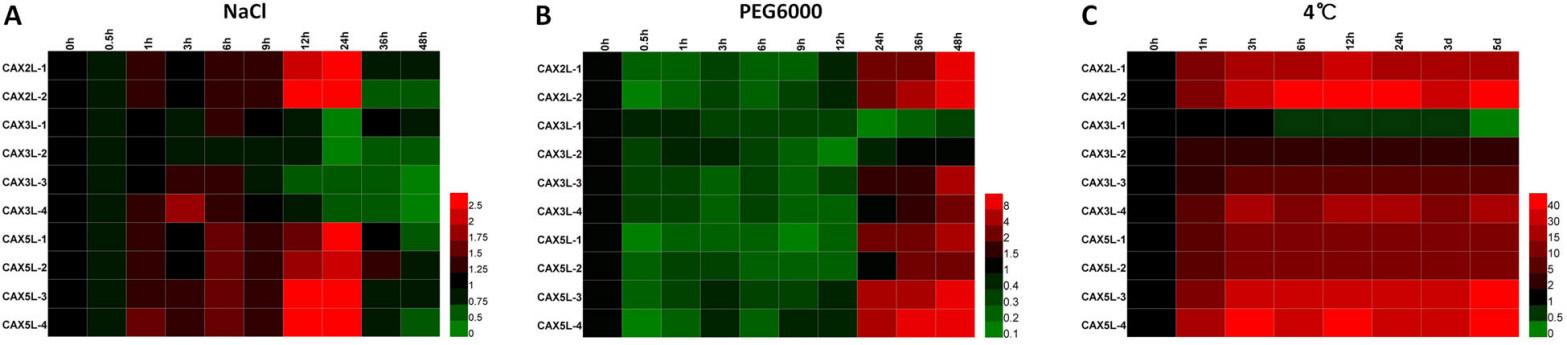
Relative expression analysis of *MdCAX* genes in apple under different abiotic stress conditions. The expression level was calculated with respect to control samples (0 h) with the 2^—△△CT^ method. Bars at the right of each heat map shows the relative expression values of these *MdCAX* genes

To further study the response of *MdCAX* genes under different abiotic stress conditions, apple hydroponic seedlings were treated with NaCl, PEG6000, and low temperature (4 °C). Under NaCl treatment, all of the genes that belonged to the Type I-B subgroup (*MdCAX2Ls* and *MdCAX5Ls*) showed an expression pattern of up-regulation followed by down-regulation (Fig. 8a). For genes that belonged to the Type I-A subgroup, the change in the expression of *MdCAX3L-1* was subtle, but *MdCAX3L-2* was down-regulated by NaCl treatment. *MdCAX3L-3* and *MdCAX3L-4* showed an expression pattern of slight up-regulation followed by significant down-regulation (Fig. 8a). The expression correlation analysis also indicated that genes belonging to the Type I-B group were highly correlated in their expression patterns (Additional file 12: Fig. S6a and Additional file 13). In group Type I-A, *MdCAX3L-1* was highly correlated with *MdCAX3L-2*, and *MdCAX3L-3* was highly correlated with *MdCAX3L-4* (Additional file 12: Fig. S6a).

To study the response of *MdCAX* genes to drought, PEG6000 (10%, W/V) was used to simulate drought treatment. Most of the *MdCAX* genes exhibited an expression pattern of down-regulation followed by up-regulation, except for *MdCAX3L-1*, which was down-regulated continuously by PEG6000 treatment (Fig. 8b). Correlation analysis showed that the expression pattern of *MdCAX3L-1* was not correlated with other genes in the *MdCAX* family (Additional file 12: Fig. S6a and Additional file 13); nevertheless, there was a high correlation between among Type I-B *MdCAX* genes. Under low temperature, most of the *MdCAX* genes were strongly up-regulated, whereas *MdCAX3L-1* exhibited a pattern of strong down-regulation (Fig. 8c). Correlation analysis also suggested that there was a negative correlation between the expression of *MdCAX3L-1* and other *MdCAXs* (Additional file 12: Fig. S6a and Additional file 13). We also evaluated the response of apple seedlings to these stress treatments by measuring the stress-related physiological parameters, including MDA and proline contents, and SOD and POD activities. The increase of these indexes indicated that the apple seedlings responded to these stress treatments significantly (Additional file 14: Fig. S7).

To further study the response of *CAX* genes to NaCl, drought, and 4 °C treatments in plants, we downloaded transcriptome data for *CAX* genes (*AtCAX1* to *AtCAX5*) in *Arabidopsis* and evaluated their expression patterns. Overall, these five *AtCAX* genes responded to different stress treatments with different expression patterns (Additional file 15: Fig. S8). The expression of genes that belonged to the Type I-B group in apple were highly correlated with the expression of *AtCAX5* under NaCl treatment (Additional file 12: Fig. S6b and Additional file 16). The expression of the four *MdCAX3L* genes in Type I-A was not highly correlated with the expression of *AtCAX1* or *AtCAX3*, but they were highly correlated with *AtCAX4* expression, a gene that also belongs to the Type I-A group (Additional file 12: Fig. S6b). Moreover, the expression of most *MdCAX3L* genes also showed a high correlation with *AtCAX4* expression under PEG treatment (Additional file 12: Fig. S6b and Additional file 16). Under 4 °C treatment, the expression of most *MdCAX* genes showed a negative correlation with *AtCAX1* or *AtCAX3* expression, whereas *MdCAX3L-1* expression was highly positively correlated with the expression of these two genes, especially with *AtCAX1* (Additional file 12: Fig. S6b).

### Prediction of the protein interaction network of CAX family proteins

**Fig. 9:**
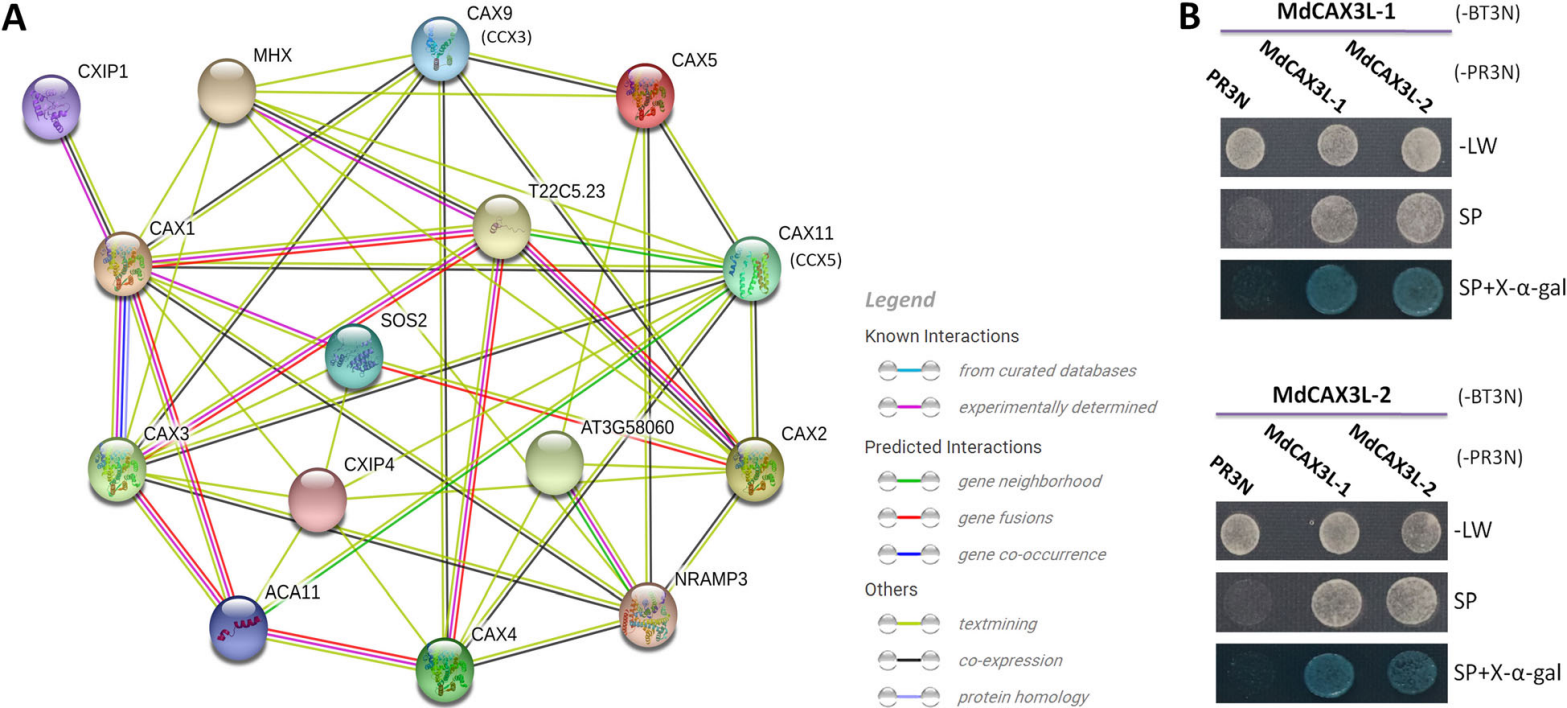
Prediction of the protein interaction network for MdCAX proteins (**a**) and verification of the interaction between MdCAX3L-1 and MdCAX3L-2 (**b**). **a** The predicted interaction network related to CAX proteins. The prediction was based on MdCAX orthologs in *Arabidopsis* using the online database STRING. Lines of different colour indicate the source of the predicted protein interaction relationships, with the purple line indicating that the interaction has been experimentally validated in *Arabidopsis*. **b** Verification of the interaction between MdCAX3L-1 and MdCAX3L-2 and between themselves by the yeast two-hybrid (Y2H) assay. BT3N and PR3N indicate the bait and prey vectors used in the Y2H assay, respectively. –LW, SD medium without leucine and tryptophan. SP (selection plates), SD medium without leucine, tryptophan, histidine, and adenine and supplemented with 2.5 mM 3-AT (3-aminotriazole)

To understand the regulatory mechanism of the abiotic stress response in these MdCAX proteins, we predicted a protein interaction network for MdCAX proteins based on their orthologs in *Arabidopsis*. Their protein sequences were first entered into the online database STRING (https://string-db.org/). After confirmation of their orthologs in *Arabidopsis* with the blastp method, a CAXs-related protein interaction network was predicted and visualised by STRING. There were complex interactions within the CAX family proteins, such as the interaction between CAX1 and CAX3 (Fig. 9a). Functional annotation indicated that these two proteins are crucial for regulating the homeostasis of ions in plants (Additional file 17: Table S4). In addition, there were also complex interactions between CAX and CCX family proteins, such as the interaction of CAX1 with CAX9 (CCX3) and CAX11 (CCX5) (Fig. 9a). In addition, we identified several proteins that play important roles in the plant stress response and that may interact with CAX proteins (Fig. 9a). For example, SOS2 is involved in regulating intracellular Na^+^ and K^+^ homeostasis and salt tolerance [51–53]. CXIP1 regulates CAX cation transporters and protects cells against protein oxidative damage. MHX, NRAMP3, and MTP8 (AT3G58060) are involved in transporting various metal ions, such as Fe, Mg, Zn, Mn, and Cd, and regulate plant metal tolerance (Additional file 17: Table S4).

Considering the high sequence similarity, the different calcium transport capacities and expression patterns of *MdCAX3L-1* and *MdCAX3L-2*, we studied the interaction between their proteins using the yeast two-hybrid membrane protein system (MoBiTec). The full-length CDS of these two genes were cloned both into the bait (pBT3N) and prey (pBR3N) vectors and were then transformed into the yeast strain NMY51 with different combinations. MdCAX3L-1 and MdCAX3L-2 interact with each other and also with themselves (Fig. 9b). This result was consistent with previous studies in *Arabidopsis* and support the predicted interaction network.

## Discussion

The CaCA superfamily has been characterised in several plant species, such as *Arabidopsis* [20, 35], rice [13], wheat [9], cotton [49], and soybean [38]. Although calcium plays an important role in stress resistance [5, 6, 11] and fruit quality [40–43, 54–56], the CaCA superfamily has not been characterised in apple. Here, we conducted the first detailed characterisation of the CaCA family in apple. Genes in the *CAX* family were cloned, and their responses to calcium and abiotic stress treatments were analyzed. By characterising calcium transport capacity and predicting the protein interaction network, this study provides a foundation for future research examining the function and mechanism of CAX proteins in regulating stress tolerance in apple.

### The expansion and evolutionary relationships of CaCA family genes in apple

Tandem and segmental duplication events are fundamental mechanisms of gene family expansion. In this study, the number of CaCA family genes in apple (Table 1) is nearly twice that in *Arabidopsis* [9]. Considering the larger genome size of apple (651 Mb) [48] relative to *Arabidopsis* (125 Mb) [57], the two genome-wide duplication events that have occurred during apple evolution [47], and the complex collinearity relationships among different apple chromosomes [47, 48], we suspect that the expansion of the CaCA family in apple is largely driven by segmental duplication. Consistent with this hypothesis, there were 14 genes (66.7%) identified that were involved in segmental duplication events (Fig. 1). Such a large proportion of genes indicates that segmental duplication was the main mode of CaCA family expansion in apple. Tandem duplication has also likely played an important role in the amplification of the CaCA family (Fig. 1). Thus, this family has expanded primarily through gene duplication events, similar to other gene families in apple [58–62].

Based on phylogenetic analysis, the 21 CaCA family proteins were divided into four groups, as in *Arabidopsis* [13]. This division of the proteins was supported by gene structure analysis and the prediction of conserved motifs (Fig. 2). Moreover, these analyses also identified several genes that differed from others within the same group, such as *MD09G1157100* (*MdCAX2L-2*), the predicted sequence of which was shown to be incorrect by gene cloning (Additional file 6: Fig. S4). The misprediction of the coding sequence was also noted for the other *MdCAX* genes (Additional file 6: Fig. S4). Thus, we suspect that other genes with special gene structures may also have sequence prediction errors, such as *MdCAX3L-5* (Fig. 2b). These errors may also explain why we were not able to obtain the CDS of this gene, using the primers that were designed according to its predicted sequence in the apple genome.

### Characterisation of calcium transport capacity of apple CAX proteins in the type I-A group

AtCAX1 and AtCAX3 are calcium transporters and also the most studied CAX family proteins in plants [13]. We thus focused on assessing the transport capacity of their homologous proteins in apple, which are named MdCAX3L-1 to MdCAX3L-4. As expected, results of the heterologous expression assay and calcium fluorescent staining assay in K667 yeast strain indicated that three MdCAX3L proteins should have a strong capacity for the transport of calcium into vacuoles (Fig. 6). However, *MdCAX3L-1* did not show any calcium ion transport capacity (Fig. 6), although its segmental duplication gene *MdCAX3L-2* did.

Sequence alignment suggested that MdCAX3L-1 contains an N-terminal autoinhibitory region (Fig. 3). Based on its inhibitory function in calcium transport among CAX proteins, we suspected that the calcium transport capacity of MdCAX3L-1 may be inhibited by this region, in a manner similar to that documented in AtCAX1 [50]. This speculation was verified in this study (Fig. 6). However, this result raises another interesting question. Given the high sequence similarity between the two segmental duplication genes *MdCAX3L-1* and *MdCAX3L-2*, why is the calcium transport capacity of MdCAX3L-2 not inhibited by this region (Fig. 6)? This same question also applies to AtCAX3 and AtCAX1 [24]. However, the sequence similarity between *AtCAX1* and *AtCAX3* was only 73.9%, and the calcium transport capacity of MdCAX3L-2 was much stronger than that of AtCAX3 [24].

Sequence alignment between MdCAX3L-1 and MdCAX3L-2 revealed that there were only three amino acid substitutions within the N-terminal autoinhibitory region and a few amino acid substitutions within the 11 transmembrane regions (Fig. 3). Based on the 3D structure and calcium ion transport mechanism of ScVCX1 [31], we hypothesised that the inhibition effect of the N-terminal autoinhibitory region was based on its interaction with specific parts of the transmembrane region. When some key amino acids within these regions changed, this interaction was disrupted, and the inhibition effect was removed. This hypothesis is consistent with the observations that deletion [2, 29, 49, 50] or interference [2] of the N-terminal autoinhibitory region can restore the transport capacity of plant CAX proteins; that SOS2 activates the transport capacity of CAX proteins by interacting with the N-terminal autoinhibitory region of CAX1 [52]; and that the maintenance of the protein structure and calcium ion transport activity of ScVCX1 was dependent on the interaction between specific amino acids within specific transmembrane regions (Figs. 3 and 4) [31]. Because most studies examining calcium transport activity of plant CAX proteins have removed the N-terminal autoinhibitory region [31, 63], these two proteins (MdCAX3L-1 and MdCAX3L-2) identified in this study could provide better models for studying the key amino acids for and the mechanisms of calcium ion transport by CAX family proteins compared with AtCAX1 and AtCAX3 [63].

### Expression analysis and prediction of the protein interaction network

CAX proteins play important roles in regulating plant resistance to various abiotic stresses [10, 11]. In this study, expression analysis showed that these *MdCAX* genes responded to calcium and abiotic stress treatments significantly (Figs. 5 and 8), which suggested that these genes also play important roles in apple stress responses. In addition, most *MdCAX* genes exhibited similar expression patterns, especially between pairs of duplication genes (Figs. 5 and 8 and Additional file 12: Fig. S6). This suggested that the duplication genes, or even genes that belonged to the same subgroup (Fig. 2), may play similar or redundant functions in apple stress responses.

Among these *MdCAX* genes, *MdCAX3L-1* showed a substantially different and even opposite expression pattern compared with other genes, especially under calcium (Fig. 5a) and cold treatments (Fig. 8c and Additional file 12: Fig. S6a). This result, combined with the difference in calcium transport capacity between MdCAX3L-1 and MdCAX3L-2 (Fig. 6) and the interaction relationship between these two proteins (Fig. 9b), suggest that these two proteins may have special regulatory mechanisms in calcium transport and the stress response. For example, they may regulate calcium transport capacity and the plant calcium signal response through different types of protein interactions. This hypothesis is consistent with the fact that the interaction between AtCAX1 and AtCAX3 promotes their calcium transport capacity significantly [24, 35].

Recent studies on the SOS2-related calcium transporter AtANN4 have proposed a feedback regulation mechanism for salt stress that dependeds on changes in the concentration of cytosolic calcium [51]. In *Arabidopsis*, AtCAX1 interacted with and was activated by AtSOS2 [52], which is crucial for resistance to salt stress [64]. Based on the predicted network (Fig. 9a), we could infer that MdCAX proteins, such as MdCAX3L-1 and MdCAX3L-2, may interact with MdSOS2, a protein that has been shown to be important for the salt tolerance in apple [65–67]. These observations further suggest that a calcium-dependent regulation mechanism might exist between the two MdCAX proteins and SOS2. In addition to SOS2, the network also identified other proteins that may interact with MdCAX proteins, such as CXIP1, MHX, NRAMP3, and MTP8 (AT3G58060) (Fig. 9), which play important roles in the stress response and its regulation (Additional file 17: Table S4). Regarding the protein interaction network is predicted based on the orthologs of MdCAXs in Arabidopsis, people should be cautious that this prediction depends on conservation of specific binding residues for both sets of proteins. This is particularly important for the residues in the N terminus of CAX proteins, because many of these CAX-interacting proteins were found to bind to the N-terminal tail domain of AtCAX1, such as SOS2 and CXIP [21, 23], and the N terminal regions of CAX proteins are typically very variable (Fig. 3). Additional research is needed to verify these predicted protein interaction relationships. This network facilitates future work aiming to characterise the mechanic role by which MdCAX proteins regulate the apple stress response.

## Conclusions

In this study, a total of 21 genes that belonging to the apple CaCA superfamily was identified from the apple genome. These genes were classified into four groups: CAX, CCX, NCL, and MHX. The exon-intron structures, conserved motif distributions, and chromosomal locations of CaCA family members in apple were also determined. Gene cloning and expression analysis revealed that *MdCAX* genes participated in the apple abiotic stress response. Prediction of the protein interaction network identified several proteins that may interact with CAX proteins and play an important role in the abiotic stress response. Combined with collinearity analysis and identification of calcium transport capacity, a pair of segmental duplication genes (*MdCAX3L-1* and *MdCAX3L-2*) exhibiting different calcium transport capacities was identified. Y2H assays suggested these two proteins can interact with each other and with themselves. Generally, these results provide a foundation for future research on *MdCAX* genes in apple.

## Methods

### Sequence retrieval and identification of apple CaCA family proteins

Apple genome-wide protein sequences (GDDH13) were downloaded from the GDR database (Genome Database for Rosaceae; https://www.rosaceae.org/), and sequences of CaCA family proteins in *Arabidopsis* were downloaded from the TAIR (The Arabidopsis Information Resource) database (https://www.arabidopsis.org/). We downloaded the HMM (Hidden Markov Model) file of the Na_Ca_ex domain (sodium/calcium exchanger protein, PF01699) from the Pfam database (Pfam 32.0; http://pfam.xfam.org/), and we used it as a query to search the apple proteome using HMMER software (version 3.1b2) with a default E-value (E-value < 0.05). The protein sequences from the HMMER screening results were then submitted to the Pfam and SMART databases (http://smart.embl-heidelberg.de) to verify the existence of the conserved Na_Ca_ex domain. Proteins that were too short (MD09G1157400, MD14G1008300) or had incomplete domains were eliminated manually (Additional file 1: Table S1).

### Chromosomal locations, collinearity analysis and characterizations of apple CaCA family genes

We downloaded the GFF file (gene_models_20170612.gff3) that contained location data for apple CaCA family genes from the GDR database. We performed a collinearity analysis between different apple chromosomes with MCScanX software. The chromosomal location and collinearity data for CaCA family genes were visualised using TBtools software. The protein length, mass weight, charge at pH 7.0, and pI (isoelectric point) value were determined with DNAstar software (version 7.1.0). The best hits in *Arabidopsis* for these apple CaCA family proteins were determined by the local blastp method with BioEdit software (version 7.0.9.0).

### Phylogenetic relationships, gene structure, and conserved motif analysis

Phylogenetic trees were constructed with MEGA-X software (version 10.0.5) using the neighbor-joining (NJ) method (parameter settings: bootstrap method, 1000 replicates; Poisson model; pairwise deletion). The intron-exon schematic structures of apple CaCA family genes were drawn with the online Gene Structure Display Server (GSDS 2.0; http://gsds.cbi.pku.edu.cn) and was based on information obtained from the GDR database (gene_models_20170612.gff3). Conserved motifs were identified using the online MEME software (5.0.2) with the following parameters: -protein -oc. -nostatus -time 18,000 -mod anr -nmotifs 25 -minw 6 -maxw 50 -objfun classic -markov_order 0.

### Sequence comparison and prediction of the three-dimensional structure of MdCAX proteins

The protein sequence of ScVCX1 was obtained from the National Center for Biotechnology Information database (NCBI, https://www.ncbi.nlm.nih.gov/), and its crystallographic structure template (4k1c.pdb) was downloaded from the Protein Data Bank database (PDB, https://www.rcsb.org/). Sequence alignment was carried out with DNAMAN software (version 6). The 3D structure of MdCAX proteins was predicted with the online software SWISS MODEL (https://swissmodel.expasy.org/) and was visualised with RasWin software (version 2.7.5.2).

### Gene cloning and expression analysis of *MdCAX* genes under calcium ion and abiotic stress treatments

To clone the full-length CDS sequcnes of the *MdCAX* genes in apple, total RNA was extracted from mature leaves of ‘Qinguan’ apple plants growing at the Horticultural Experimental Station of Northwest A&F University (Yangling, Shaanxi, China). Experiments that involved calcium or abiotic stress treatments were conducted in a controlled environment chamber. For expression analysis of *MdCAX* genes under different treatments, tissue-cultured apple seedlings (‘Royal Gala’, GL-3) were used. Before treatments, seedlings were transferred to rooting medium (MS + 0.1 mM IAA) for rooting. One month later, plantlets with a consistent growth state were selected and transferred into hydroponic conditions (Hoagland nutrient solution) for 10 days. These plantlets were then selected again and treated with calcium (100 mM CaCl_2_), NaCl (200 mM), PEG6000 (10%; W/V), or low temperature (4 °C) for various periods under continuous white light conditions. The light intensity was set to 10,000 lx, and the humidity was set to 70%. For calcium, NaCl, and PEG6000 treatments, the temperature was set to 25 °C. RNA extraction and real-time quantitative RT-PCR analysis followed previously described methods [62]; gene-specific primers are listed in Additional file 18. Heatmaps were drawn with HemI software (Illustrator for heatmap, Version 1.0).

For expression analysis of *AtCAX* genes under different abiotic stress conditions, the GEO datasets for *Arabidopsis* were downloaded from the NCBI database (salt-GSE5623, drought-GSE5624, cold-GSE5621). The intergroup correlation analysis among *MdCAX* genes and the intra-group correlation analysis between *MdCAX* and *AtCAX* genes were conducted with the online platform OmicShare (https://www.omicshare.com/tools/) using Spearman correlation analysis.

### Calcium ion treatment and calcium ion fluorescence staining of yeast strain k667

To obtain the yeast strain transformed with different *MdCAX* genes, the four *MdCAX* genes (*MdCAX3L-1* to *MdCAX3L-4*) and the truncated *MdCAX3L-1* were cloned into the yeast expression vector pDR-196 and then transferred into the calcium ion-sensitive yeast mutant strain k667. After selection on SD medium (−ura) and PCR screening for the presence of the transgene, more than two monoclonal strains of each *MdCAX* gene were obtained and were used for subsequent Ca^2+^ treatments.

For treatments using different concentrations of calcium ions, control yeast strain (K667) or K667 strains transformed with different *MdCAX3L* genes were cultured in liquid YPD medium to a concentration of OD_600_ = 1.0. The bacterial solutions were then diluted four times in a 10-fold gradient and placed on solid YPD or YPD medium varying in the concentration of CaCl_2_. After three days, the growth of the strains was observed and photographed.

The calcium-sensitive fluorescent probe Fluo4-AM was used to observe Ca^2+^ levels inside yeast cells transformed either with pDR196 (empty vector) or with different *MdCAX* genes. Before fluorescence observations, yeast cells were grown on liquid SD medium (−ura) to the exponential phase and then diluted to OD_600_ = 0.2 ~ 0.3 in medium supplemented with 50 mM CaCl_2_. After 4 h of culture at 30 °C, yeast cells were washed with PBS solution three times to remove the medium and were then incubated with 5 μM Fluo4-AM for 20 min at 30 °C. Subsequently, the yeast cells were washed three times with PBS to remove the fluorescent dye, and the fluorescence was observed using a laser scanning confocal microscope (Leica TCS SP8 SR). The excitation and emission wavelengths for GFP fluorescence were set to 488 nm and 512–520 nm, respectively. The LAS AF software (Leica Application Suite Advanced Fluorescence, version 4.3) was used to determine the grey values of green fluorescence in vacuoles of yeast cells.

### Promoter analysis and prediction of the protein interaction network

To analyze the promoter of *MdCAX* genes, the apple genome sequence (GDDH13_1-1_formatted.fasta) was downloaded from the GDR database, and sequences 1500 bp in length upstream of the transcription start site (ATG) of these *MdCAX* genes were extracted. These sequences were then entered into the online program PlantCARE (http://bioinformatics.psb.ugent.be/webtools/plantcare/html/) to search for cis-acting elements related to stress responsiveness and plant hormones. The protein interaction network of CAX proteins was predicted using the online database STRING (version 11.0; https://string-db.org/), with the organism specified as ‘*Arabidopsis thaliana*’.

### Yeast two-hybrid (Y2H) assay

Because the CAXs were membrane proteins, the yeast two-hybrid membrane protein system (MoBiTec) was used. The full-length CDS of *MdCAX3L-1* and *MdCAX3L-2* were cloned into both the bait vector pBT3-N and the prey vector pPR3-N (MoBiTec) respectively, using the One Step Cloning Kit (Vazyme) per the manufacturer’s instructions. These constructs were then transformed into the yeast strain NMY51 using a lithium acetate method per the manufacturer’s instructions (MoBiTec). Yeast cells were spread on SD medium (−Leu/−Trp) for culture, and positive clones were further confirmed by PCR screening. The transformed colonies were then plated onto select medium (SD-Leu/−Trp/−His/−Ade supplemented with 2.5 mM 3-AT) with or without X-α-gal (20 μg/ml) to test for possible interactions.

## Supplementary Information

**Additional file 1: Table S1.** HMMER screening results and conservative domain comparison between CaCA family proteins in apple and *Arabidopsis*.

**Additional file 2.** Sequences of the proteins from HMMER screening results.

**Additional file 3: Fig. S1.** Phylogenetic analysis of putative CaCA family proteins in apple and CaCA family proteins in *Arabidopsis*. Red dots indicate proteins that may not belong to the CaCA family and needed to be removed in subsequent analysis.

**Additional file 4: Fig. S2.** The conserved domain analysis of NCL proteins in apple and *Arabidopsis*. The protein sequences of AtNCL and four MdNCL proteins were entered into the SMART database to search for conserved domains.

**Additional file 5: Fig. S3.** Putative conserved motifs identified in the sequences of apple CaCA family proteins.

**Additional file 6: Fig. S4.** Sequence comparison between the predicted *CAX* genes in the apple genome and the *CAX* genes that were actually cloned in this study.

**Additional file 7 **The CDS and protein sequences of the cloned *MdCAX* genes in this study.

**Additional file 8: Table S2.** Basic information for the 3D structure prediction of MdCAX proteins in apple.

**Additional file 9. **Sequences of the promoter regions (− 1500 bp) of *MdCAX* genes.

**Additional file 10: Table S3.** Cis-elements identified in the promoter regions of *MdCAX* genes in apple. Plus and minus signs indicate whether the cis-element was located either the plus or minus strand.

**Additional file 11: Fig. S5.** Cis-elements identified in the promoter regions of *MdCAX* genes in apple.

**Additional file 12: Fig. S6.** Correlation analysis between the expression patterns of *MdCAX* genes in apple (A) or between *MdCAX* and *AtCAX* genes (B).

**Additional file 13. **The *p*-value matrix of the intragroup correlation analysis among *MdCAX* genes.

**Additional file 14: Fig. S7.** MDA and proline contents and enzyme activities of SOD and POD. Bars labelled with different letters indicate signigicant differences at *P* < 0.05 based on one-way ANOVA and Duncan’s tests (SPSS software, version 26).

**Additional file 15: Fig. S8.** The expression patterns of *AtCAX* genes under abiotic stress conditions in *Arabidopsis*.

**Additional file 16 **The *p*-value matrix of the intergroup correlation analysis between *MdCAX* and *AtCAX* genes.

**Additional file 17: Table S4.** Functional annotation of CAXs and their interacting proteins.

**Additional file 18. **qRT-PCR primers of *MdCAX* genes used in this study.

## Data Availability

We uploaded the nucleotide sequences of the cloned *MdCAX* genes to GenBank with the accession numbers MT820134 for *MdCAX2L-1*, MT820135 for *MdCAX2L-2*, MT820136 for *MdCAX3L-1*, MT820137 for *MdCAX3L-2*, MT820138 for *MdCAX3L-3*, MT820139 for *MdCAX3L-4*, MT820140 for *MdCAX5L-1*, MT820141 for *MdCAX5L-2*, MT820142 for *MdCAX5L-3*, and MT820143 for *MdCAX5L-4*. The remaining data used in this study are included in the article and its additional files.

## Abbreviations

pI: Isoeletric point
CDS: Coding sequence
UTR: Untranslated region
ABA: Abscisic acid
MeJA: Methyl jasmonate
GA: Gibberellin
SA: Salicylic acid
NJ: Neighbor joining
qRT-PCR: Quantiative real-time reverse transcription PCR; PBS: Phosphate buffer saline; Y2H Yeast two-hybrid
3-AT: 3-aminotriazole
X-α-gal: 5-bromo-4-chloro-3-indolyl-α-D-galactopyranoside.
