# Supplementary figures and images for: Genome-wide analysis of the apple CaCA superfamily reveals that MdCAX proteins are involved in the abiotic stress response as calcium transporters
Source: BMC Plant Biol. 2021 Feb 8;21:81. doi: 10.1186/s12870-021-02866-1 (PMC7869505; doi:10.1186/s12870-021-02866-1)

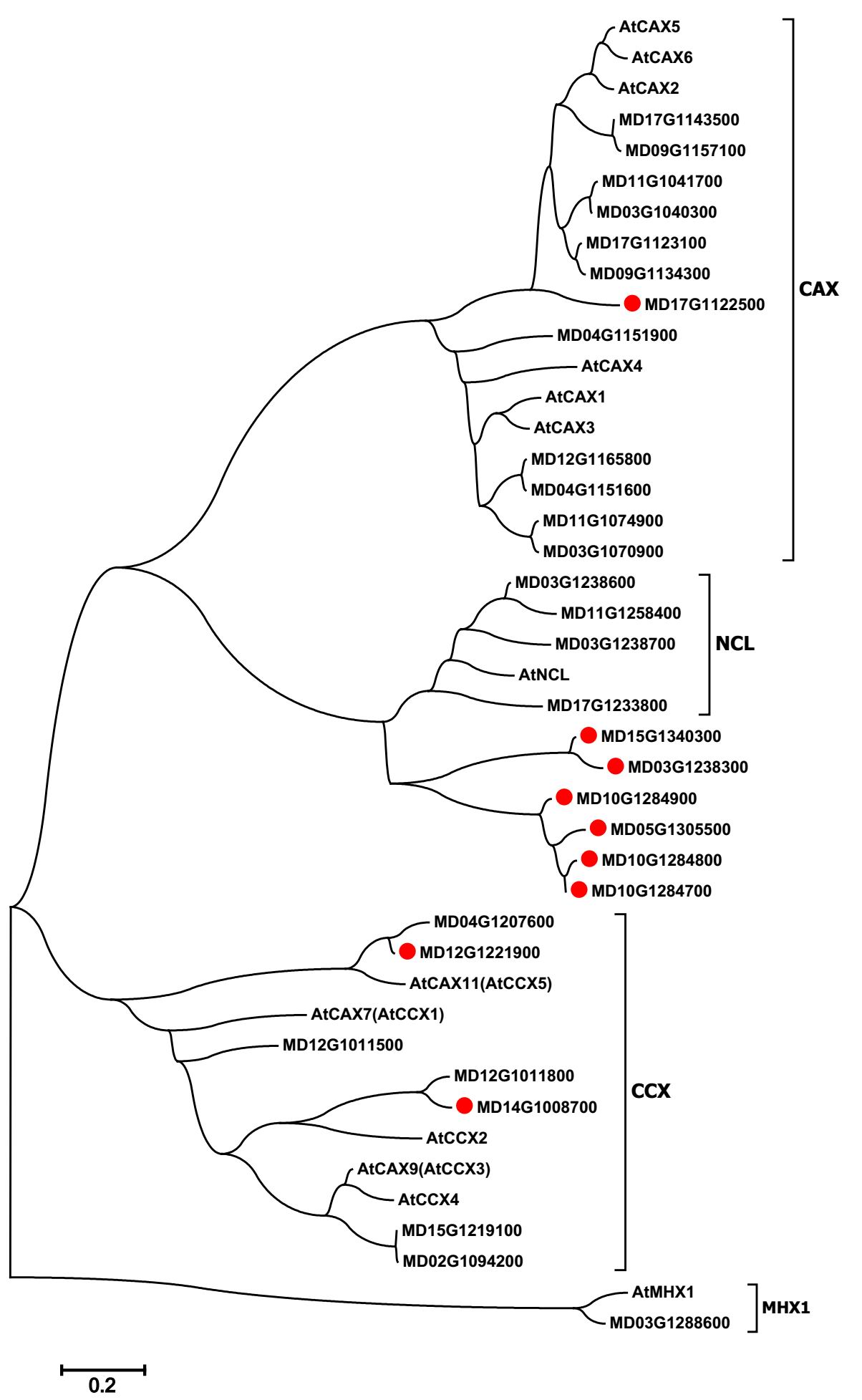

Supplement: Supplementary file 3 — Additional file 3: Fig. S1. Phylogenetic analysis of putative CaCA family proteins in apple and CaCA family proteins in Arabidopsis. Red dots indicate proteins that may not belong to the CaCA family and needed to be removed in subsequent analysis. [file 12870_2021_2866_MOESM3_ESM.jpg]

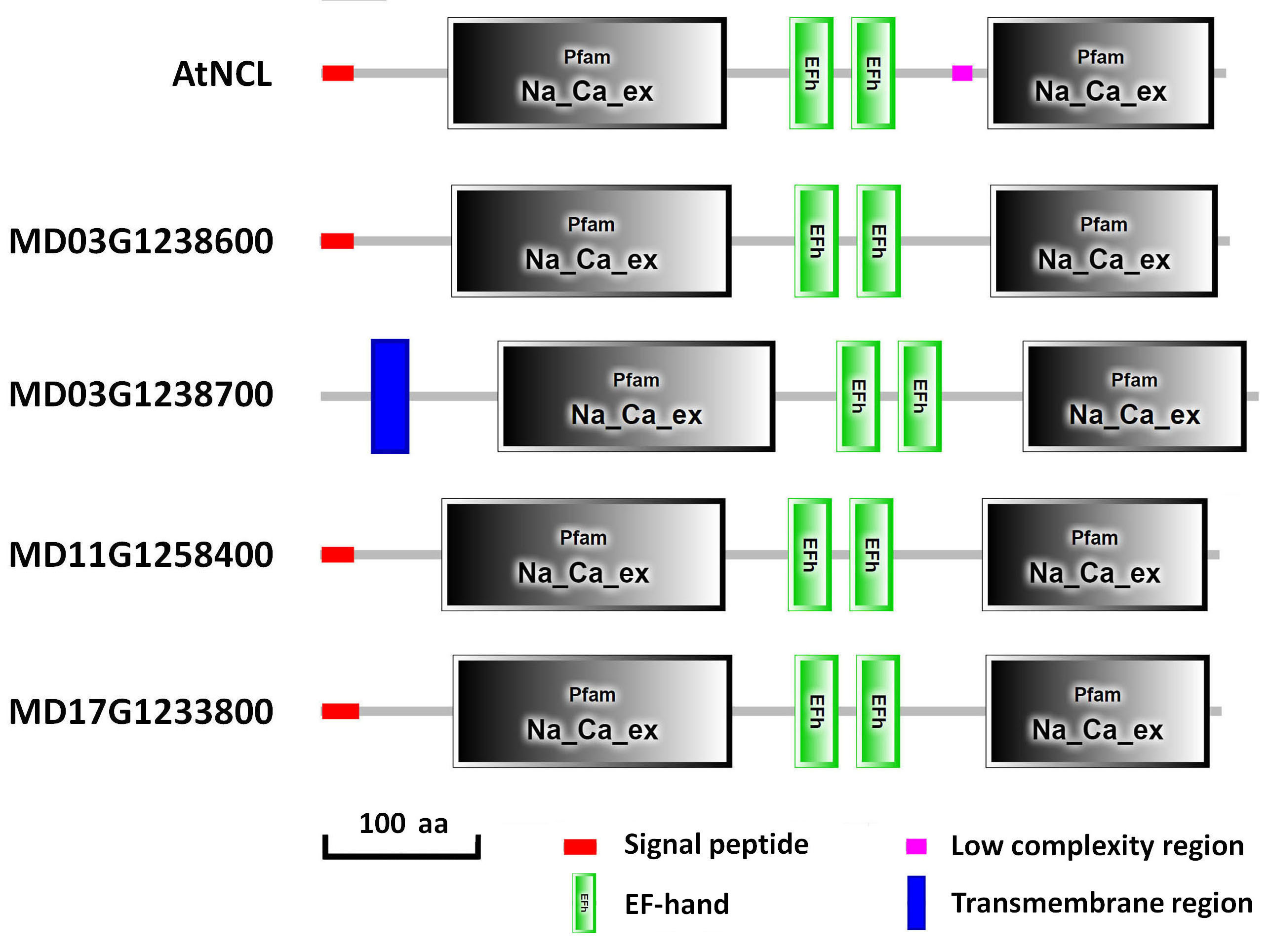

Supplement: Supplementary file 4 — Additional file 4: Fig. S2. The conserved domain analysis of NCL proteins in apple and Arabidopsis. The protein sequences of AtNCL and four MdNCL proteins were entered into the SMART database to search for conserved domains. [file 12870_2021_2866_MOESM4_ESM.jpg]

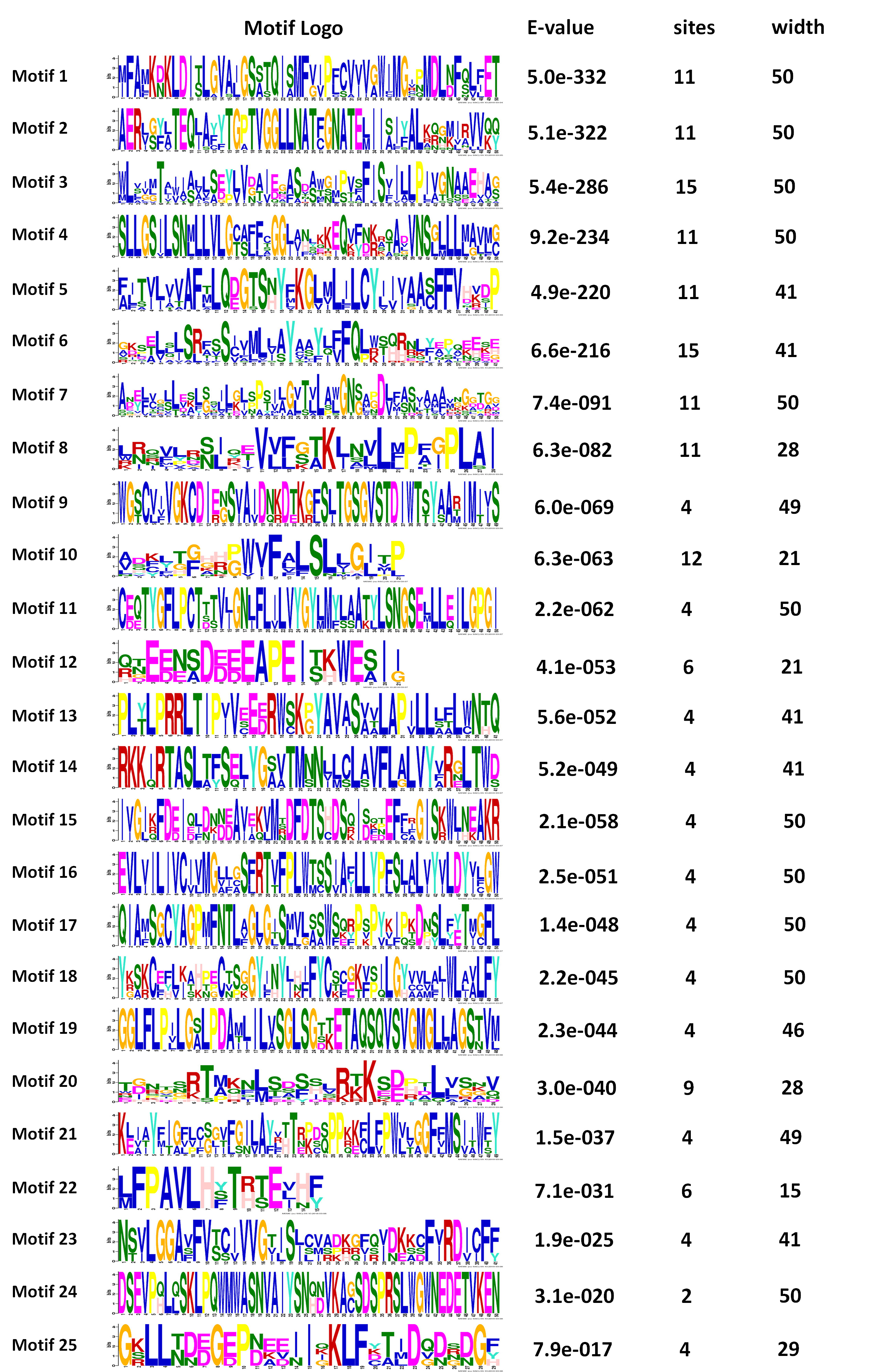

Supplement: Supplementary file 5 — Additional file 5: Fig. S3. Putative conserved motifs identified in the sequences of apple CaCA family proteins. [file 12870_2021_2866_MOESM5_ESM.jpg]

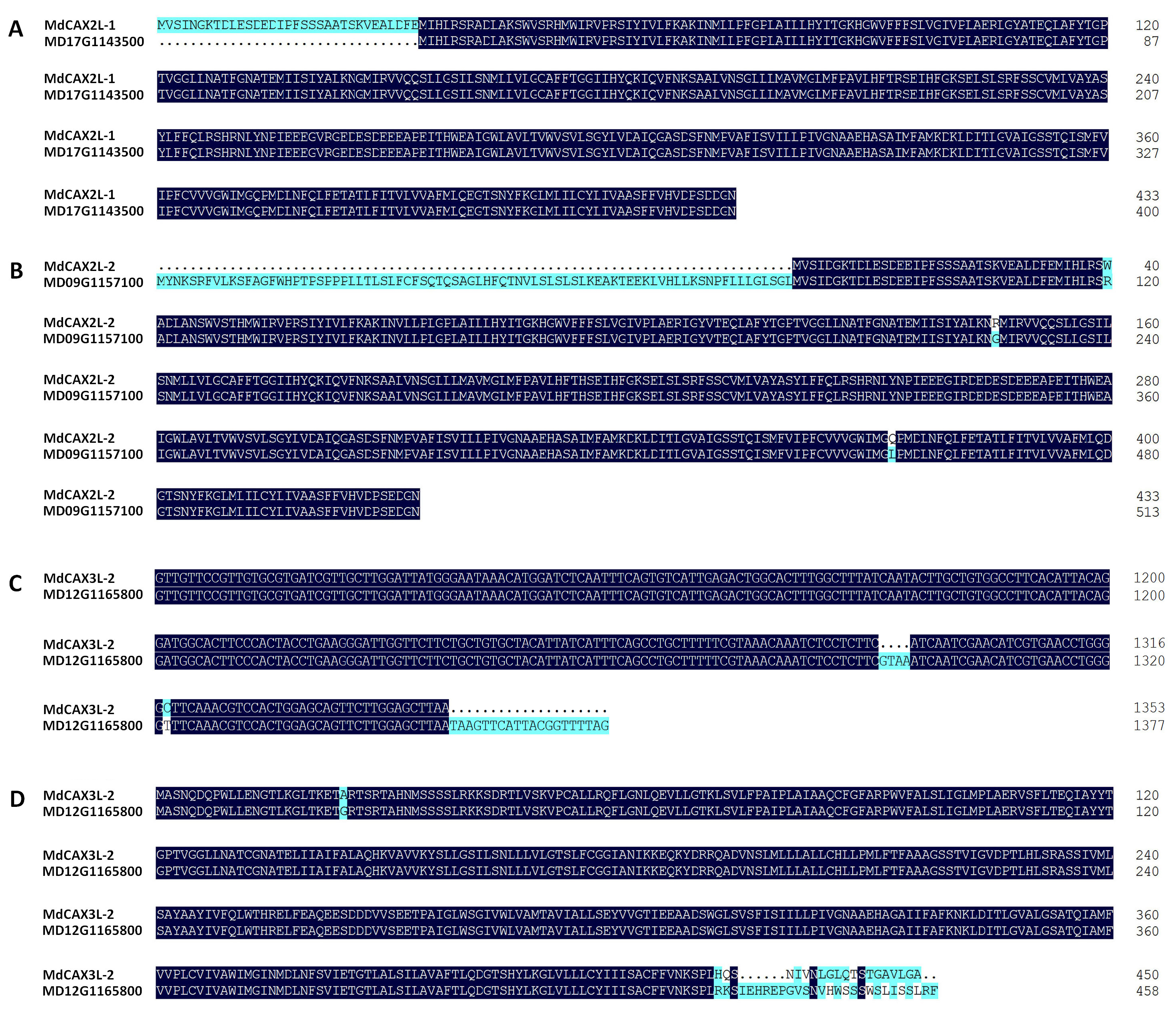

Supplement: Supplementary file 6 — Additional file 6: Fig. S4. Sequence comparison between the predicted CAX genes in the apple genome and the CAX genes that were actually cloned in this study. [file 12870_2021_2866_MOESM6_ESM.jpg]

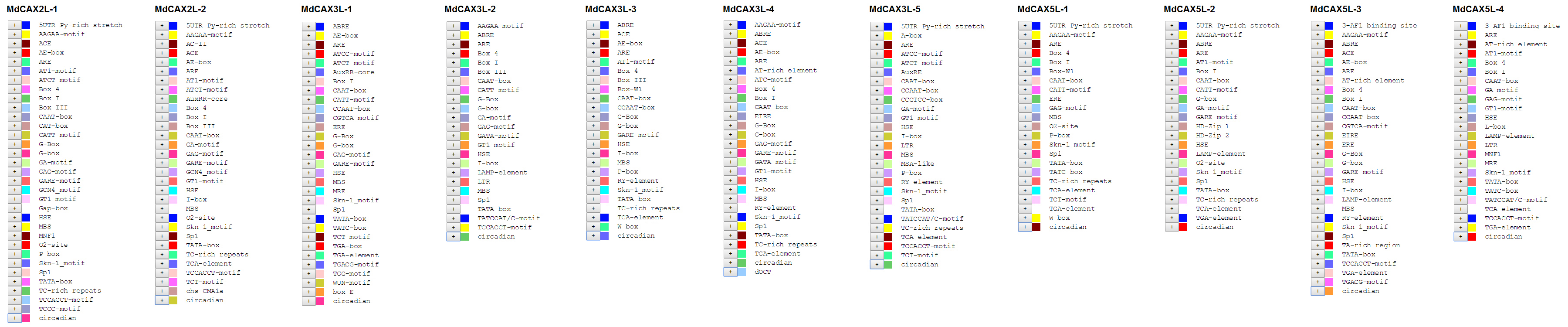

Supplement: Supplementary file 11 — Additional file 11: Fig. S5. Cis-elements identified in the promoter regions of MdCAX genes in apple. [file 12870_2021_2866_MOESM11_ESM.jpg]

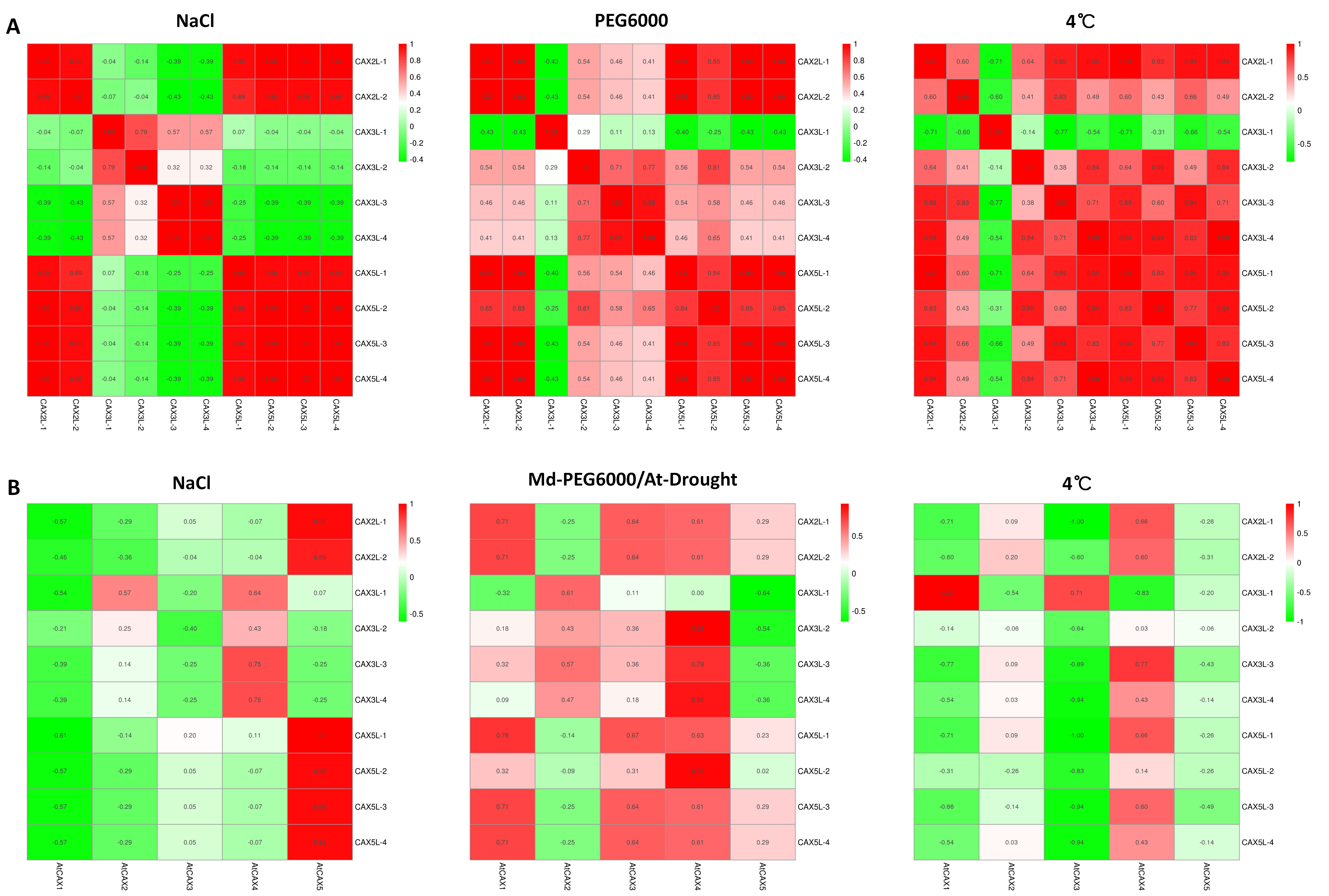

Supplement: Supplementary file 12 — Additional file 12: Fig. S6. Correlation analysis between the expression patterns of MdCAX genes in apple (A) or between MdCAX and AtCAX genes (B). [file 12870_2021_2866_MOESM12_ESM.jpg]

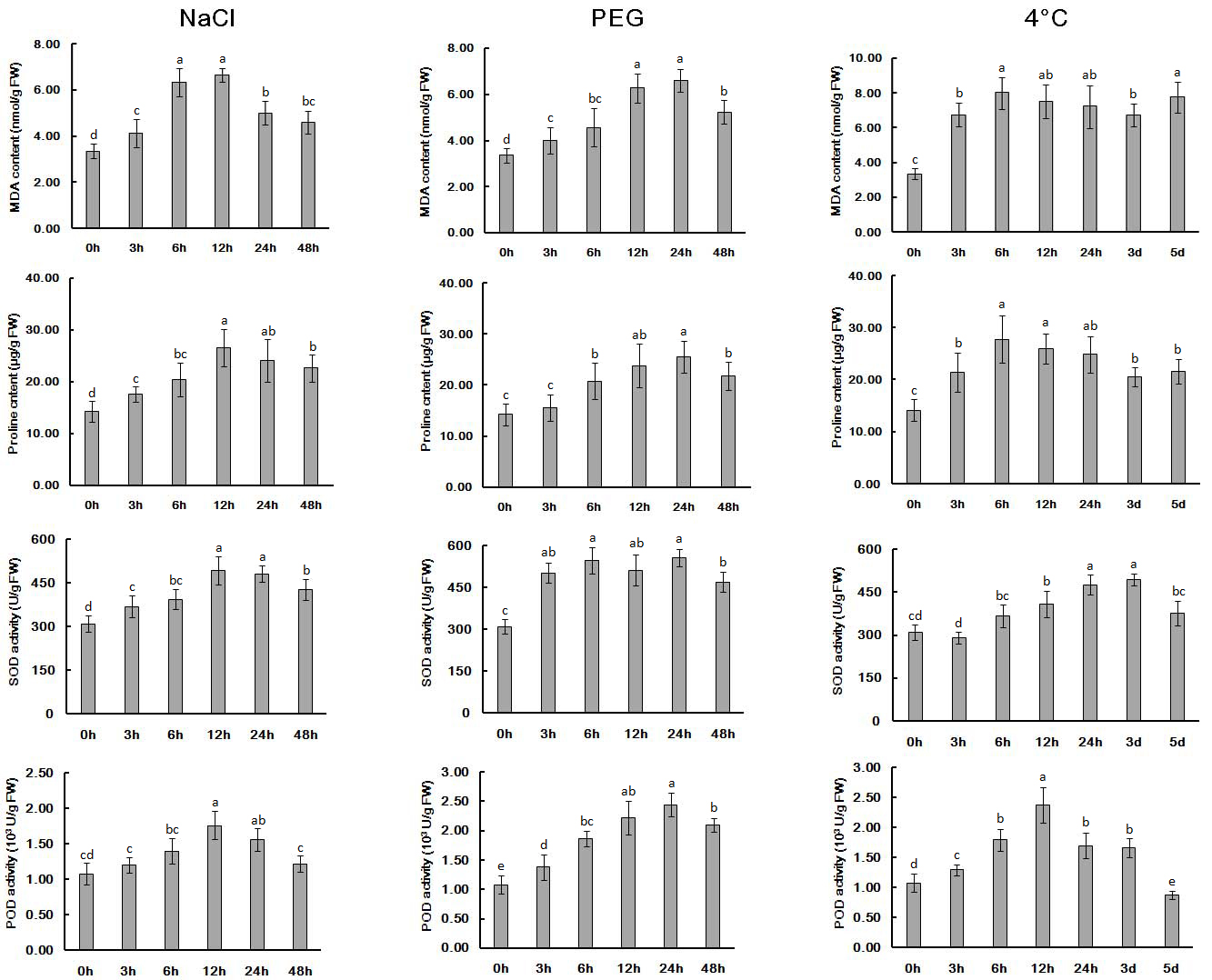

Supplement: Supplementary file 14 — Additional file 14: Fig. S7. MDA and proline contents and enzyme activities of SOD and POD. Bars labelled with different letters indicate signigicant differences at P < 0.05 based on one-way ANOVA and Duncan’s tests (SPSS software, version 26). [file 12870_2021_2866_MOESM14_ESM.jpg]

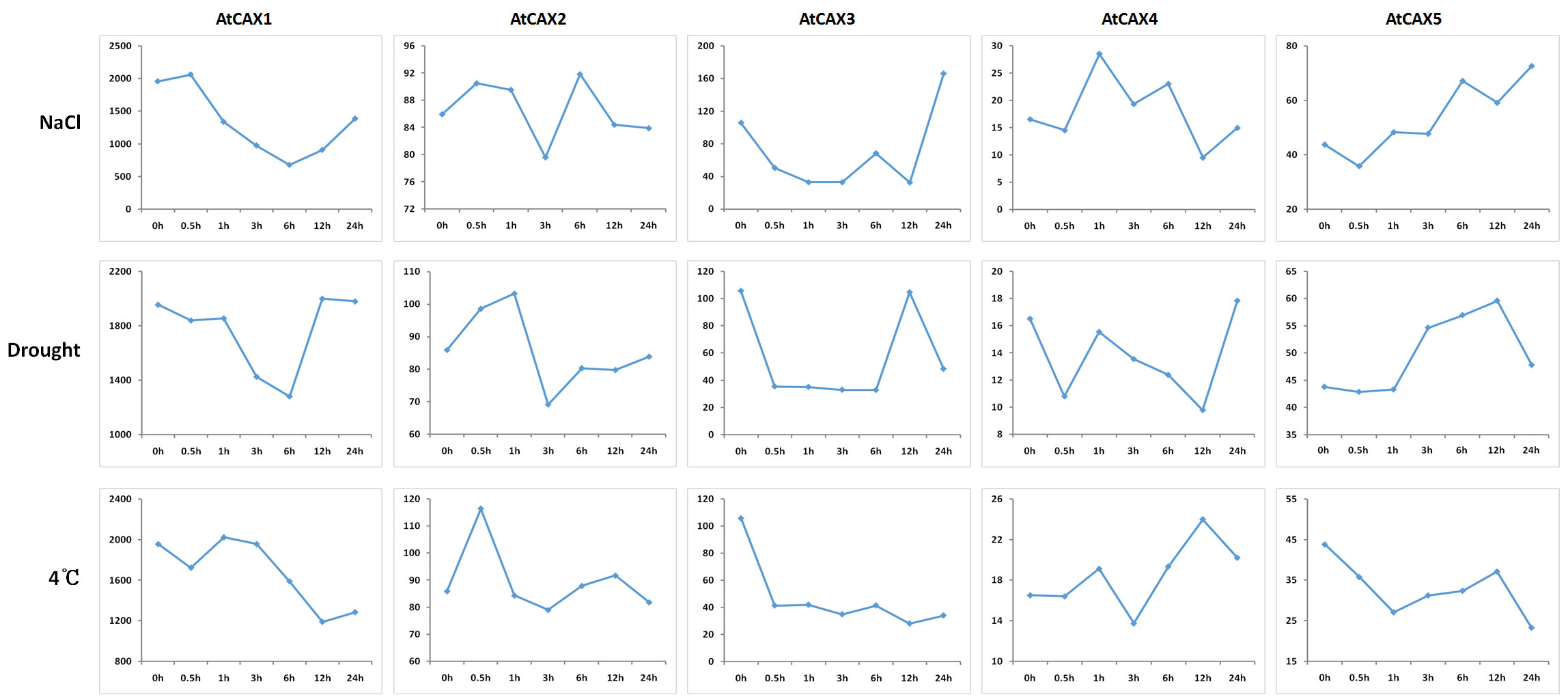

Supplement: Supplementary file 15 — Additional file 15: Fig. S8. The expression patterns of AtCAX genes under abiotic stress conditions in Arabidopsis. [file 12870_2021_2866_MOESM15_ESM.jpg]
